# Ccdc94 Protects Cells from Ionizing Radiation by Inhibiting the Expression of *p53*

**DOI:** 10.1371/journal.pgen.1002922

**Published:** 2012-08-30

**Authors:** Shelly Sorrells, Seth Carbonneau, Erik Harrington, Aye T. Chen, Bridgid Hast, Brett Milash, Ujwal Pyati, Michael B. Major, Yi Zhou, Leonard I. Zon, Rodney A. Stewart, A. Thomas Look, Cicely Jette

**Affiliations:** 1 Department of Oncological Sciences, Huntsman Cancer Institute, University of Utah, Salt Lake City, Utah, United States of America; 2 Department of Pediatric Oncology, Dana-Farber Cancer Institute and Harvard Medical School, Boston, Massachusetts, United States of America; 3 Department of Hematology/Oncology, Children's Hospital, Boston, Massachusetts, United States of America; 4 Department of Cell and Developmental Biology, Lineberger Comprehensive Cancer Center, Chapel Hill, North Carolina, United States of America; 5 Bioinformatics Shared Resource, Huntsman Cancer Institute, University of Utah, Salt Lake City, Utah, United States of America; University of Washington, United States of America

## Abstract

DNA double-strand breaks (DSBs) represent one of the most deleterious forms of DNA damage to a cell. In cancer therapy, induction of cell death by DNA DSBs by ionizing radiation (IR) and certain chemotherapies is thought to mediate the successful elimination of cancer cells. However, cancer cells often evolve to evade the cytotoxicity induced by DNA DSBs, thereby forming the basis for treatment resistance. As such, a better understanding of the DSB DNA damage response (DSB–DDR) pathway will facilitate the design of more effective strategies to overcome chemo- and radioresistance. To identify novel mechanisms that protect cells from the cytotoxic effects of DNA DSBs, we performed a forward genetic screen in zebrafish for recessive mutations that enhance the IR–induced apoptotic response. Here, we describe *radiosensitizing mutation 7* (*rs7*), which causes a severe sensitivity of zebrafish embryonic neurons to IR–induced apoptosis and is required for the proper development of the central nervous system. The *rs7* mutation disrupts the coding sequence of *ccdc94*, a highly conserved gene that has no previous links to the DSB–DDR pathway. We demonstrate that Ccdc94 is a functional member of the Prp19 complex and that genetic knockdown of core members of this complex causes increased sensitivity to IR–induced apoptosis. We further show that Ccdc94 and the Prp19 complex protect cells from IR–induced apoptosis by repressing the expression of *p53* mRNA. In summary, we have identified a new gene regulating a dosage-sensitive response to DNA DSBs during embryonic development. Future studies in human cancer cells will determine whether pharmacological inactivation of CCDC94 reduces the threshold of the cancer cell apoptotic response.

## Author Summary

Radiation therapy and most chemotherapies elicit cancer cell death through the induction of excessive DNA damage. However, cancer cells can harbor genetic defects that confer resistance to these therapies. To identify cellular components whose targeted therapeutic inactivation could potentially enhance the sensitivity of treatment-resistant cancer cells to DNA–damaging therapies, we have chosen an unbiased genetic approach in live whole zebrafish embryos to identify genes that normally protect cells from the lethal effects of DNA damage. This approach has yielded the discovery of a novel radioprotective gene called *ccdc94*. Upon inactivation of *ccdc94*, cells become more sensitive to radiation-induced cell death. Our further analysis revealed that the Ccdc94 protein functions in the Prp19 complex, which is known to regulate gene expression and repair of damaged DNA. We found that this complex normally represses radiation-induced cell death by inhibiting the expression of the *p53* gene, a critical mediator of DNA damage–induced cell death. Future experiments that inactivate Ccdc94 and Prp19 complex proteins in human cancer cells will determine if inactivation of this complex represents a novel therapeutic strategy that could increase *p53* expression to enhance sensitivity to DNA damaging therapies in chemo- and radio-resistant cancer cells.

## Introduction

After cells undergo genotoxic stress, multiple DNA-damage response (DDR) pathways are essential for the faithful replication and transmission of chromosomes to subsequent generations. Depending on the type of lesion, different pathways are engaged to repair the DNA [1]. One of the most detrimental lesions to occur upon exposure to ionizing radiation (IR) and certain chemotherapies is the DNA double-stranded break (DSB). Immediate cell cycle arrest following DNA DSBs plays a critical role in promoting efficient DNA repair before cells enter mitosis. When exposed to excessive amounts of DNA DSBs that overwhelm their repair machinery, cells that are competent to do so will undergo p53-dependent apoptosis [2]. While the exact events that determine how this decision is made are not well understood, it is clear that p53-mediated transcriptional induction of the BH3-only protein Puma is critical for IR-induced apoptosis [3]–[5]. Puma induction triggers the activation of Bax and Bak [6] leading to mitochondrial outer membrane permeabilization, release of apoptotic factors including cytochrome C, and activation of the Caspase cascade of proteolytic degradation. Once Caspases are activated, an irreversible program of cellular destruction ensues. Anti-apoptotic members of the Bcl-2 family of proteins, like Bcl-2 and Bcl-xL, can inhibit this process by binding and sequestering Puma (and other BH3-only proteins) to prevent activation of Bax/Bak. Consequently, mutations that lead to the overexpression of Bcl-2 or to the impairment of the p53 pathway play pivotal roles not only in the development and progression of cancer, but also in the resistance to chemo- and radiotherapy that develops in established tumors [2], [7].

Interestingly, a number of genes with prominent functions in the DSB-DDR pathway are also required for normal development of the nervous system [8]. Ataxia-Telangiectasia (A–T) was one of the earliest recognized diseases that arise from defects in the DSB-DDR pathway and is characterized by severe ataxia, radiosensitivity, defective immune function, sterility and predisposition to cancer [9]. A–T is caused by homozygous recessive mutations in *ataxia-telangiectasia mutated* (*ATM*) [10], a gene encoding a kinase that plays pivotal roles in sensing DNA DSBs and coordinating a complex cellular signaling response that mediates the commitment to undergo cell cycle arrest, DNA repair and apoptosis [11]. Developing neurons are highly proliferative and the associated increase in oxidative stress likely exposes them to excessive DNA damage, thereby explaining their unique sensitivity to defects in the DSB-DDR pathway [8]. Since p53-dependent apoptosis is a common consequence of excessive DNA damage in this tissue [2], developing neurons are selectively dosage-sensitive to IR. Indeed, we and others have shown that the developing nervous system in zebrafish represents an excellent system to identify genes required for the DSB-DDR [3]–[4], [12].

The DSB-DDR pathway is complex and remains incompletely understood [13]. ENU-based genetic screens provide an unbiased method for identifying new components of the DSB-DDR pathway whose inactivation could kill cells that have become resistant to DNA-DSB-inducing therapies. To date, there are no published accounts of forward genetic screens performed in vertebrate *in vivo* models designed to identify novel radioprotective genes. Here we describe a rapid, thirty-hour zebrafish screen to identify mutations that enhance apoptosis after exposure to moderate levels of IR. One of the mutants we identified from this screen, which we named *radiosensitizing mutation 7* (*rs7*), causes a severe sensitivity of zebrafish embryonic neurons to IR-induced apoptosis and is required for the proper development of the central nervous system. Hypersensitivity to DNA-DSBs by *rs7* arises from an increase in *p53* mRNA expression and activity. We have mapped the *rs7* mutation to an early stop codon within *ccdc94*, a gene that encodes a protein with few known functions or informative domains but that is highly conserved from yeast to humans [14]. Using biochemical and genetic approaches, we demonstrate that Ccdc94 is a functional component of the Prp19 complex in vertebrate cells. Prp19 complex members have established roles in pre-mRNA splicing [15]–[16] and DNA repair [17]. We show that depleting components of this complex renders cells more sensitive to DNA damage because of inappropriately high *p53* mRNA and protein levels, but this effect does not arise from a global splicing defect. In summary, by taking advantage of the powerful embryonic and genetic attributes of the zebrafish system, we have identified a new gene regulating a dosage-sensitive DSB-DDR pathway during development.

## Results

**Figure 1:**
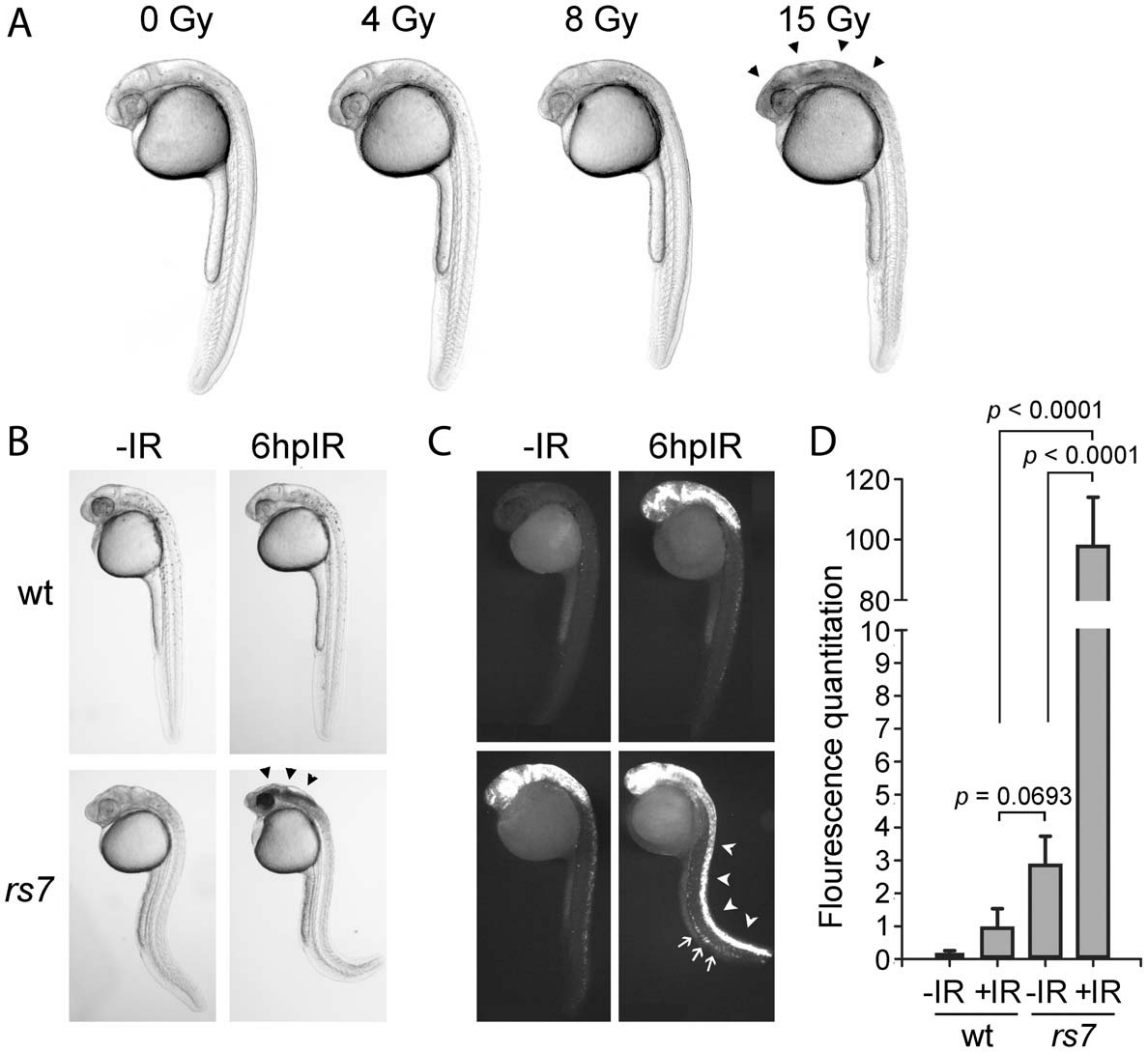
Identification of *radiosensitizing mutation #7*. (A) Wild-type AB strain embryos were exposed to the indicated amount of IR at 24 hpf and visualized by brightfield microscopy 6 hours later. A threshold of IR exists between 8 and 15 Gy that gives rise to obvious cell death (seen as opaque tissue, marked by arrowheads) in the brain. (B) Wild-type embryos (derived from crossing wild-type parents) or *rs7* mutant embryos (derived from crossing *rs7* heterozygous parents) were exposed to 8 Gy IR at 24 hpf and visualized by brightfield microscopy 6 hours later. Arrowheads in the *rs7* mutant mark cell death reminiscent of exposure of wild-type embryos to 15 Gy (as shown in A) (C) Embryos in (B) were sorted by phenotype, fixed at 6 hpIR and analyzed by immunofluorescence to detect activated Caspase-3. The “curly-up” tail phenotype and opaque tissue in the head were used to identify *rs7* mutants. These phenotypes are present (to different degrees of severity) in both unirradiated and irradiated mutants such that they can be readily distinguished from siblings and wild-type at 30 hpf. After fixation of mutants, tails were clipped to distinguish them from wild-type embryos, which were analyzed in the same tube for Caspase-3 activity. Embryos were grouped for analysis according to whether embryos were irradiated or not, and *rs7* mutants were identified based on the presence of tail-clips. Arrowheads point to the enhanced apoptosis in the spinal cord of an *rs7* mutant, and arrows point to increased apoptosis in the ICM region of an *rs7* mutant. (D) Immunofluorescence in the spinal cords from at least 10 embryos per group in (C) was quantified. wt; wild-type. See also Figure S1.

To discover novel radioprotective genes using the zebrafish genetic model, we first sought to identify an obvious bright-field phenotype in embryos that distinguishes different levels of apoptotic response to IR. High doses of IR, such as 15 Gy, administered to a transparent zebrafish embryo at 24 hours post-fertilization (hpf) cause extensive apoptosis in neural tissue resulting in the accumulation of opaque tissue in the head. This phenotype is very consistent and readily observable by bright-field microscopy by six hours post-IR (hpIR, Figure 1A, arrowheads). We treated wild-type zebrafish embryos with different levels of IR and found that the opaque tissue in the head was not observed using doses less than or equal to 8 Gy. These data show that a threshold of IR exposure exists between 8 and 15 Gy in wild-type embryos that gives rise to the obvious opaque neural tissue phenotype. We then reasoned that any mutation that inactivates a radioprotective gene should sensitize the embryonic neural tissue such that exposure of embryos to 8 Gy would cause a phenotype reminiscent of 15 Gy.

Based on this logic, we performed a recessive genetic screen using 8 Gy IR (Figure S1) and identified a number of mutations that sensitize embryos to IR. The first mutation we characterized was *rs7* (Figure 1B) because these mutants showed a pronounced response to 8 Gy IR. The *rs7* mutation gives rise to a homozygous recessive radiosensitive phenotype. Embryos that are heterozygous for the *rs7* mutation are phenotypically identical to homozygous wild-type embryos with reference to all of the assays performed in this study. To verify that the observed neural cell death in irradiated *rs7* mutants was due to apoptosis, we fixed the embryos at six hpIR and performed immunofluorescence with an antibody to detect activated Caspase-3 (Figure 1C). Wild-type embryos showed moderate levels of activated-Caspase-3-positive apoptotic cells in the brain and spinal cord in response to 8 Gy IR. By comparison, irradiated *rs7* mutants showed a dramatic increase in cell death in the neural tissue when compared to wild-type irradiated embryos (Figure 1C, arrowheads). We also occasionally noticed an increase in IR-induced apoptosis in the intermediate cell mass (ICM) where primitive hematopoietic tissue resides (Figure 1C, arrows), but we remained focused on analyzing the consistent neural radiosensitive phenotype. Unirradiated *rs7* mutant embryos exhibited a level of apoptosis in neural tissue that appeared similar to that of irradiated wild-type embryos indicating that the *rs7* mutation also causes apoptosis in neural tissue independent from IR. For clarity, this will subsequently be referred to as “*rs7*-mediated neurodegeneration.”

Since radiosensitization is characterized by a multiplicative, rather than additive, effect on the response to IR, we questioned whether the enhanced apoptosis in the irradiated *rs7* mutants represented a true radiosensitization or simply an *addition* of *rs7*-mediated neurodegeneration to the apoptosis that is normally induced by 8 Gy IR. To address this question, we quantified levels of fluorescence in each of the four experimental groups from Figure 1C and plotted the values in Figure 1D, normalizing the response of irradiated wild-type embryos to one. As suggested by Figure 1C, unirradiated *rs7* mutants showed levels of fluorescence that were not significantly different from those of irradiated wild-type embryos. Notably, compared to irradiated wild-type embryos, irradiated *rs7* mutants have a 95-fold increase in activated-Caspase-3 staining. These data show that *rs7* is a *bona fide* radiosensitizing mutation.

While identification of mutations like *rs7* that cause neurodegeneration is a relatively common event in zebrafish ENU-mutagenesis screens [18]–[19], we found that only 11% (4/36) of the neurodegenerative mutants we identified also gave rise to a radiosensitizing phenotype. Conversely, we have also identified radiosensitizing mutations that do not affect the survival of neural tissue at 30 hpf (our unpublished data). Indeed, morpholino knockdown of known components of the DSB-DDR pathway also leads to radiosensitization of neural tissue with or without associated developmental neurodegeneration ([20]–[21], our unpublished observations). This suggests that sensitivity to IR is not a frequent consequence of compromised survival in neural cells.

**Figure 2:**
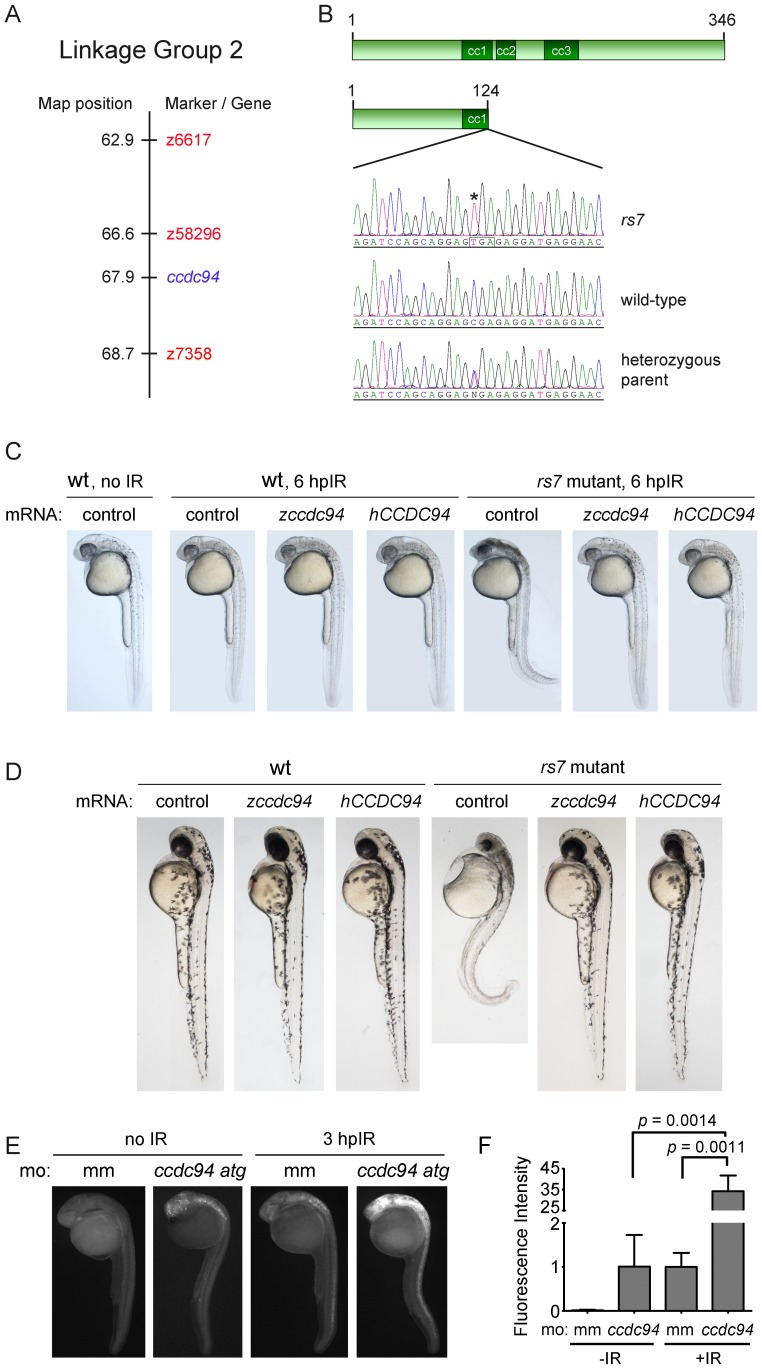
The *rs7* phenotype is caused by a mutation in the *ccdc94* gene. (A) The *rs7* mutation was localized to a 2.1 cM region (compared to the Massachusetts General Hospital genetic map for linkage group 2) as defined by flanking markers z58296 and z7358. (B) Sequencing of candidate genes (based on gene expression profiles) revealed a premature stop codon in the *ccdc94* gene. Sequence from the *ccdc94* gene exon 4 is shown for an *rs7* mutant, an unrelated wild-type AB embryo, and a heterozygous parent of the *rs7* mutant. Asterisk indicates the *rs7* point mutation, and box illustrates mutation to a TGA nonsense codon. (C) Wild-type AB strain or *rs7* heterozygous fish were incrossed, and the progeny were injected with mRNA encoding the indicated genes at the one-cell stage of development. At 24 hpf, embryos were exposed to 8 Gy IR and visualized by brightfield microscopy 6 hours later. (D) Embryos were injected similar to (C) but were not irradiated. Instead, they were left to develop until 48 hpf. (C and D) Because *rs7* mutant and sibling embryos injected with wild-type *ccdc94* mRNAs were morphologically indistinguishable, pictures were taken first, and the embryos were subsequently genotyped to identify mutants. (E) Wild-type embryos were injected with 400 µM mismatch morpholino (mm) as a negative control, or a translation-blocking *ccdc94* morpholino (*ccdc94 atg*). Embryos were then irradiated at 24 hpf with 8 Gy IR and analyzed three hours later by immunofluorescence to detect activated Caspase-3. (F) Embryos in (E) were quantified similar to Figure 1D. Comparisons between mismatch morpholino-injected embryos plus and minus IR generated a *p* value of 0.0114. See also Figures S2 and S3.

Genetic linkage analysis of the *rs7* phenotype to 239 microsatellite markers revealed that markers z58296 and z7358 flanked a 2.1 centimorgan region on chromosome 2 that harbored the *rs7* mutation (Figure 2A). Only 40 genes were annotated between these markers in the available zebrafish genome assembly Zv7 (http://www.ensembl.org/Danio_rerio), and none of these genes were implicated in the DSB-DDR response pathway. We reasoned that the gene harboring the *rs7* mutation should be expressed in the brain and spinal cord because these tissues were selectively radiosensitized in *rs7* mutants. Using published gene expression data for the 40 genes in the interval, we narrowed our list of candidates to six genes based on high levels of RNA expression in neural tissues (http://zfin.org/cgi-bin/webdriver?MIval=aa-pubview2.apg&OID=ZDB-PUB-040907-1). We sequenced five of these genes and found a premature stop codon in *coiled-coil domain containing gene 94* (*ccdc94*, R125Stop, Figure 2B).

The *Ccdc94* gene is present in genomes from yeast to humans (http://www.ensembl.org/Danio_rerio/Gene/Summary?g=ENSDARG00000026185r=2:52746237-52760438) and has previously been shown to regulate pre-mRNA splicing [14]. While the zebrafish and human protein sequences share 67% overall identity (Figure S2), the first 175 amino acids are nearly an exact match (94% identity). The Ccdc94 protein contains three predicted coiled-coil domains (http://www.ensembl.org/Danio_rerio/Transcript/Summary?g=ENSDARG00000026185r=2:52746237-52760438t=ENSDART00000036813), all of which would be either eliminated or disrupted by the R125Stop mutation, suggesting that the R125Stop mutation interrupts a highly conserved function of Ccdc94.

To determine if the R125Stop mutation caused the *rs7* radiosensitivity phenotype, we tested whether wild-type *ccdc94* mRNA could rescue the excessive IR-induced apoptosis in *rs7* mutants. We injected one-cell stage wild-type embryos, or embryos derived from a cross between *rs7* heterozygotes, with mRNAs encoding either zebrafish *ccdc94,* human *CCDC94* or *egfp* (control). We irradiated the clutches with 8 Gy and found that both zebrafish *ccdc94* and human *CCDC94* mRNA rescued the *rs7* bright-field radiosensitization phenotype (Figure 2C). We also followed the development of *rs7* mutant embryos in the absence of IR and found that the *rs7* mutation causes major deterioration of neural tissue resulting in a small head and curled tail phenotype by day 2 (Figure 2D, Figure S3) and death by the end of day 3. To prove that this phenotype was also caused by the mutation in *ccdc94*, we performed the rescue experiment described above, but instead of irradiating the embryos at 24 hpf, we allowed them to develop unperturbed until 48 hpf. Figure 2D demonstrates that both zebrafish *ccdc94* and human *CCDC94* mRNA rescued the *rs7* morphological phenotype at 48 hpf. Ultimately, however, this transient rescue (injected mRNA generally lasts for up to three days) did not rescue these embryos from lethality, as they eventually succumbed to complications from developing edema and overall dysmorphic effects by day 6 (data not shown). Nonetheless, these data demonstrate that the early embryonic radiosensitizing and neurodegenerative phenotypes of *rs7* mutants are specifically due to the loss of a highly conserved function of Ccdc94.

To independently show that loss of the *ccdc94* gene causes the *rs7* phenotype, we knocked-down the endogenous *ccdc94* in wild-type animals with an anti-sense translation-blocking morpholino. Figure 2E–2F demonstrates that *ccdc94* knockdown radiosensitizes embryos as measured by whole-mount immunofluorescence to detect activated Caspase-3. Since the *ccdc94* knockdown only increases Caspase-3 activity by 34-fold (compared to 95-fold in *rs7* mutants, Figure 1D), the morpholino likely induces a partial knockdown of Ccdc94. While the “curly-up” phenotype seen in the *rs7* mutants (Figure 1B, Figure 2C–2D) is not obvious at 27 hpf in *ccdc94* morphants, it becomes prominent by 2 days-post-fertilization (data not shown). As such, we have confirmed by three independent assays that the *rs7* phenotype is due to the effects of a recessive loss-of-function mutation in the *ccdc94* gene.

The anti-apoptotic oncoprotein Bcl-2 has been shown in a number of studies to confer cancer-cell resistance to IR and chemotherapy [22]–[24]. To determine whether the *rs7* mutation could overcome *bcl-2* overexpression and restore apoptosis after exposure to IR, we injected one-cell stage wild-type or *rs7* mutant embryos with 5 pg of *bcl-2* mRNA. Figure S4 shows that while 5 pg of *bcl-2* mRNA completely abolishes all apoptosis in irradiated wild-type embryos, it cannot achieve the same response in *rs7* mutant animals, which exhibit typical levels of IR-induced apoptosis despite overexpression of *bcl-2* mRNA. However, since *rs7* mutants injected with 5 pg of *bcl-2* mRNA exhibit *less* apoptosis than control-injected *rs7* mutants (Figure S4), we hypothesized that higher levels of *bcl-2* expression would fully block *rs7*-mediated radiosensitivity. Indeed, injection of 50 pg of *bcl-2* mRNA (or mRNA encoding *bcl-xL*, another anti-apoptotic member of the Bcl-2 family) was able to completely block the *rs7*-mediated radiosensitization. These experiments indicate that Ccdc94 is a dose-dependent modifier of the anti-apoptotic function of *bcl-2* in a manner that is genetically upstream of *bcl-2* in the DSB-DDR pathway.

**Figure 3:**
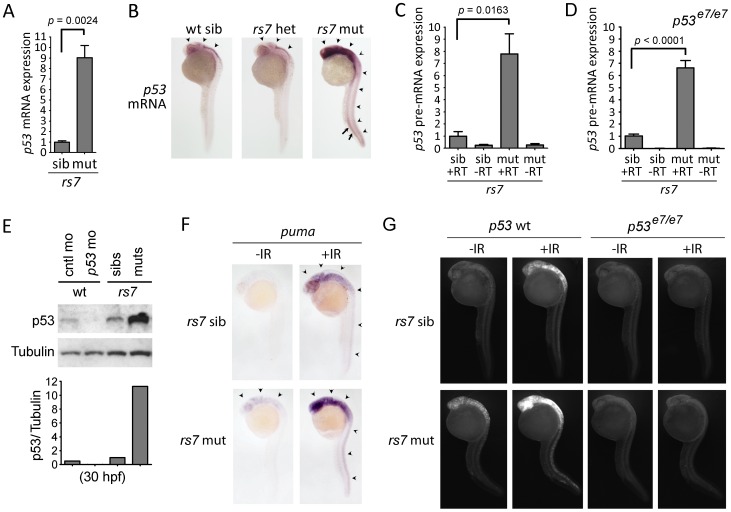
The *rs7*-mediated radiosensitizing phenotype is caused by an increase in *p53* mRNA expression. (A) Thirty hpf *rs7* siblings and mutants were identified based on morphology (similar to 1C). RNA was isolated, reverse transcribed using oligo-dT primers and analyzed for the expression of *p53* mRNA by qPCR. Expression of the *gapdh* gene was also analyzed to normalize *p53* mRNA levels. All data was then compared to sibling data, which was adjusted to a value of one. (B) *rs7* siblings and mutants were grown to 24 hpf and analyzed by whole-mount *in situ* hybridization with a probe complementary to *p53* mRNA. High levels of *p53* mRNA expression are evident in neural tissue (arrowheads) and the ICM (arrows). Pictures were taken first, and the embryos were subsequently genotyped to identify mutants and heterozygous or wild-type siblings. (C) RNA from (A) was reverse transcribed with random hexamers, and intron 9 of the *p53* gene was analyzed by qPCR to determine levels of *p53* pre-mRNA. Expression of *28S* RNA was also analyzed to normalize *p53* pre-mRNA levels. Similar results were obtained from an analysis of intron 4 (data not shown). All data was then compared to sibling data, which was adjusted to a value of one. (D) *rs7^+/−^;p53^e7/e7^* fish were incrossed to analyze *rs7* siblings and mutants in a *p53* homozygous mutant background. *Rs7* mutants and siblings were distinguished by morphology since loss of p53 does not prevent the *rs7*-mediated “curly-up” tail phenotype. RNA was harvested at 30 hpf and analyzed as in (C). (E) Protein was harvested from *rs7* siblings and mutants, and p53 and control morphants (injected at 400 µM) at 30 hpf and analyzed for p53 and Tubulin (as a loading control). ImageJ software was used to quantify band intensity from film. Shown below the blot are values for p53 divided by values for Tubulin (corresponding to above lanes) with *rs7* siblings normalized to one. (F) *rs7* sibling or mutant embryos were irradiated at 24 hpf with 8 Gy IR and analyzed 2 h later by whole-mount *in situ* hybridization with a probe complementary to *puma* mRNA. Arrowheads point to *puma* expression in neural tissue. (G) *rs7* sibling or mutant embryos in the *p53* wild-type or homozygous mutant background were irradiated at 24 hpf with 8 Gy IR and analyzed three hours later by immunofluorescence to detect activated Caspase-3. For panels (A), (C), and (D), error bars represent the standard error of the mean from at least three independent experiments. Panels (B) and (F–G) show representative data from at least three independent experiments. RT; reverse transcriptase. See also Figures S4, S5, S6.

Since the only described function for Ccdc94 involved the regulation of splicing in yeast [14], we investigated if it played a similar role in vertebrates. We sequenced the transcriptome of 30 hpf wild-type, *rs7* siblings and *rs7* mutants by Illumina RNA-Seq analysis and compared gene expression profiles. We found no obvious global differences in splicing since greater than 96% of genes showed no significant differences (i.e., *p*>0.05) in mRNA expression, and there was no evidence of changes to alternative splicing. Instead we found a remarkable increase in *p53* mRNA levels that were 3.4 and 5.1-fold higher in *rs7* mutants compared to *rs7* siblings and wild-type embryos, respectively. These results were confirmed by quantitative real-time reverse transcriptase PCR (qPCR) and whole-mount *in situ* hybridization with a probe complementary to *p53* mRNA (Figure 3A, 3B). Interestingly, the high expression of *p53* in both the neural and hematopoietic tissue in *rs7* mutants (Figure 3B) mirrors the high expression of *ccdc94* in these same tissues in wild-type embryos (http://zfin.org/cgi-bin/webdriver?MIval=aa-pubview2.apg&OID=ZDB-PUB-040907-1 and data not shown). To determine if elevated *p53* mRNA levels were likely due to either increased *p53* transcription or enhanced mRNA stabilization, we measured levels of intronic *p53* sequence as a representation of pre-mRNA. Indeed, *rs7* mutants show a similar increase in *p53* pre-mRNA (Figure 3C) suggesting that the elevated levels of *p53* are likely due to increased transcription.

Robu *et al* [25] have demonstrated the activation of a p53-dependent “general stress response” driven by morpholino off-target effects. While this pathway likely required the post-translational activation of p53, rather than upregulation of *p53* transcription, we were concerned that induction of full-length *p53* mRNA in *rs7* mutants could be a downstream feed-forward mechanism of the p53-dependent general stress response. We tested this possibility by performing the same experiments in a *p53* mutant background (*p53^e7/^* ^e7^, [26]) to eliminate the transcriptional activity of the p53 protein. Analysis of *rs7;p53* double mutants showed that *p53* mRNA remains highly elevated in this context and is not simply an indirect consequence of a general p53-dependent stress response (Figure 3D).

We next sought to clarify whether the increased *p53* mRNA in *rs7* mutants was due to specific regulation of *p53* transcription or a general activation of the DSB-DDR pathway. To answer this question, we first tested wild-type embryos for IR-mediated induction of *p53* mRNA. We found that IR exposure in wild-type embryos leads to an increase in *p53* mRNA expression (Figure S5A) that is modest compared to unirradiated *rs7* mutants (Figure 3A). We next analyzed the *rs7* mutants for upregulation of genes that are known to be induced by IR-induced activation of E2F1 in a p53-independent manner, such as *apaf1*, *caspase7*, and *p73* [27]. To measure p53-independent gene expression, we performed the experiment in the *p53^e7/^* ^e7^ background. Figure S5B demonstrates that none of these genes are significantly upregulated by the *rs7* mutation. These experiments suggest that the *rs7* mutation specifically induces *p53* expression through a selective mechanism that is not simply due to general activation of the DSB-DDR pathway.

To determine whether the increased *p53* mRNA in *rs7* mutants translated to increased p53 protein, we analyzed protein levels in *rs7* siblings and mutants by western analysis using a previously described antibody to zebrafish p53 (ZFp53-9.1, [28]). Indeed, we found that the *rs7* mutation caused a dramatic increase in p53 protein compared to siblings (Figure 3E). A previously characterized *p53* morpholino [26] was included to demonstrate antibody specificity. RNA-Seq analysis showed that a reduction in Mdm2 expression was likely not contributing to increased p53 protein levels since *mdm2* mRNA levels were 1.5- and 1.2-fold higher in *rs7* mutants than in *rs7* siblings and wild-type embryos, respectively. These experiments define a new role for Ccdc94 in embryonic development as a negative regulator of *p53* mRNA and protein expression.

We next questioned whether an increase in p53 protein activity accounts for the extreme radiosensitivity of the *rs7* mutants to IR. Since *p53*-dependent *puma* induction is essential for IR-induced apoptosis in mammals and zebrafish [4]–[5], [29], we analyzed expression of *puma* mRNA as a measure of p53 transcriptional activity. As expected, after exposure to IR, *puma* expression is much stronger in *rs7* mutants than siblings (Figure 3F), and the expression of *puma* in irradiated mutants and siblings requires wild-type *p53* (data not shown). To validate the requirement for *p53* in *rs7*-mediated radiosensitization, we tested the *rs7* mutation in the *p53* mutant background. We irradiated embryos at 24 hpf, analyzed them three hours later for apoptosis, and found that wild-type *p53* is required to execute the *rs7*-mediated radiosensitivity (Figure 3G). These experiments suggest that the *rs7*-dependent increase in p53 protein and IR-induced activity accounts for the *rs7*-mediated radiosensitivity of developing neural tissue.

In the *absence* of IR, *rs7* mutants show increased *puma* expression in neural tissue (Figure 3F, arrowheads) compared to siblings, a sign that increased *p53* mRNA expression translates to an increase in p53 pro-apoptotic activity even in the absence of an exogenous DNA-damage signal. Overexpression of *puma* has been shown to have a potent pro-apoptotic effect in zebrafish embryos [3], [12], so we questioned whether this might contribute to the developing neurodegenerative phenotype that becomes strikingly evident by day 2 in *rs7* mutants (Figure 2D, Figure S3 (arrowheads)). We found that expression levels of *puma* were extremely high in *rs7* mutants as measured at 32 hpf and 48 hpf by qPCR, and this aberrant expression was entirely dependent on the presence of wild-type *p53* (Figure S6A). In *p53* mutants, where *puma* expression was absent, there was a marked reduction in neural cell death and an obvious improvement in brain development in *rs7* mutants by brightfield microscopy (Figure S6B, arrows). While loss of wild-type *p53* prolonged the life of *rs7* mutants by 2–3 days, it ultimately failed to rescue the developmental lethality (data not shown) indicating that Ccdc94 also has essential p53-independent roles in development. However, these data show that inactivation of p53 significantly rescues the developmental neurodegeneration in *rs7* mutants, and the pro-apoptotic activity of Puma likely contributes to *rs7*-mediated neurodegeneration.

**Figure 4:**
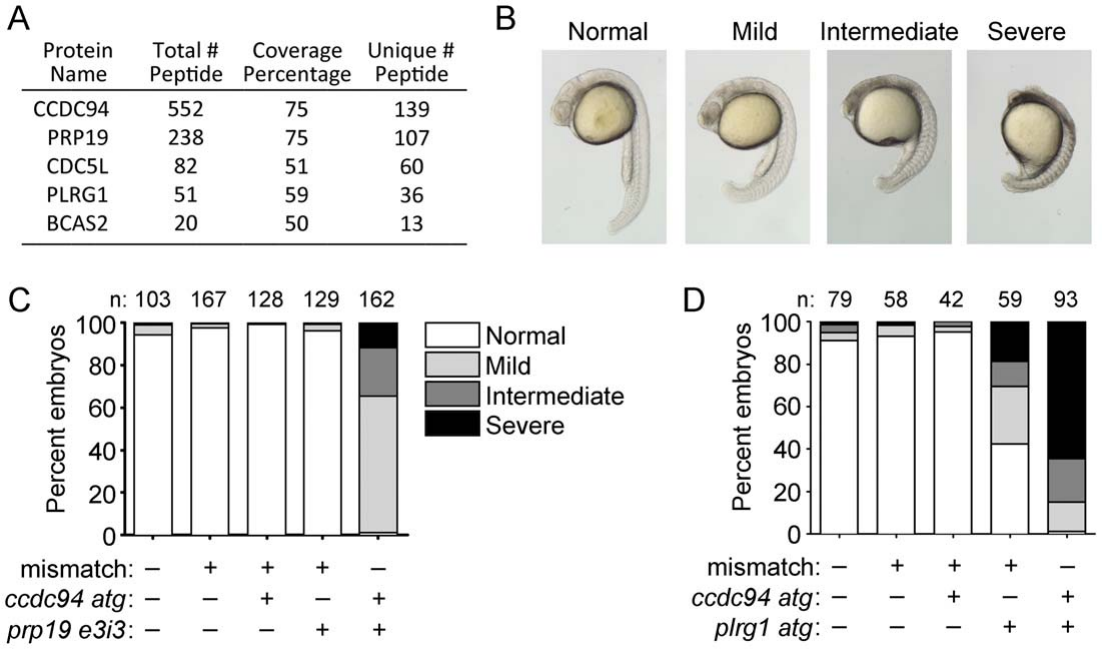
Ccdc94 interacts with core members of the Prp19 complex. (A) TAP/MS analysis shows that human CCDC94 binds to core members of the PRP19 complex. “Total Peptide #” indicates the number of peptides present in the analysis that are derived from the indicated protein. “Coverage Percentage” refers to the percent of protein sequence for a given protein that is represented by the peptides. “Unique # Peptide” refers to the number of unique peptides found for the indicated protein. (B, C) Wild-type embryos were injected with the *ccdc94 atg* morpholino (400 µM), *prp19 e3i3* morpholino (200 µM), or mismatch morpholino (as a negative control and to keep the total concentration of morpholino injections at 600 µM) and grown to 22 hpf. A range of phenotypes were observed in the embryos as shown in (B). Embryos showing the different phenotypes from (B) were quantified in (C). (D) Wild-type embryos were injected with the *ccdc94 atg* morpholino (400 µM), *plrg1 atg* morpholino (20 µM), or mismatch morpholino (as a negative control and to keep the total concentration of morpholino injections at 420 µM), grown to 22 hpf, and analyzed as in (C). Data in (C) and (D) is derived from one representative experiment; however, three independent experiments were qualitatively similar for each panel. Number (n) of embryos analyzed for each group is indicated. See also Figure S7.

To gain further insight into the molecular function of Ccdc94 in the DSB-DDR pathway, we performed a tandem-affinity purification followed by mass spectrometry (TAP/MS) of human CCDC94 in mammalian cells. Specifically, *CCDC94* cDNA was cloned into the pGlue vector encoding a dual-affinity tag containing streptavidin-binding protein, calmodulin-binding protein, and the hemagglutinin epitope. Lines of 293T cells expressing low levels of the tagged-bait fusion proteins were generated, detergent-solubilized, subjected to two rounds of affinity purification, trypsinized and analyzed by liquid chromatography–tandem mass spectrometry. The top hit was CCDC94 (Figure 4A) which serves as a positive control for the analysis. Interestingly, some of the other highly significant hits (PRP19, CDC5L, PLRG1, BCAS2) are core members of the PRP19 complex [30]–[32] which has previously been shown to be required for pre-mRNA splicing in yeast [15]–[16] and for DNA repair in both yeast and human cells [33]–[37].

As an independent validation that Ccdc94 interacts with Prp19 complex members, we analyzed whether *ccdc94* could genetically interact with *prp19* or *plrg1 in vivo*. We reasoned that targeting each gene by morpholino knockdown would allow us to titrate levels of each protein to best reveal a potential genetic interaction. We designed a splice-blocking morpholino targeting *prp19* (called *prp19 e3i3*, Figure S7) and made use of a previously characterized translation-blocking *plrg1* morpholino [38]. We injected the highest dose of each morpholino that gave rise to minimal apoptosis or developmental abnormalities. At 22 hpf, injection with *ccdc94* or *prp19* morpholino alone gave rise to mostly normally developing embryos whereas the *plrg1* morpholino alone led to an accumulation of cell death in the central nervous system in about half of injected embryos with varying severity (represented in Figure 4B and quantified in Figure 4D), similar to *plrg1* mutants [39] and previously characterized morphants [38]. Strikingly, these same developmental abnormalities were highly abundant among embryos co-injected with *ccdc94* morpholino and either *prp19* or *plrg1* morpholinos (Figure 4B–4D). These experiments indicate that *ccdc94* genetically interacts with both *prp19* and *plrg1 in vivo*.

**Figure 5:**
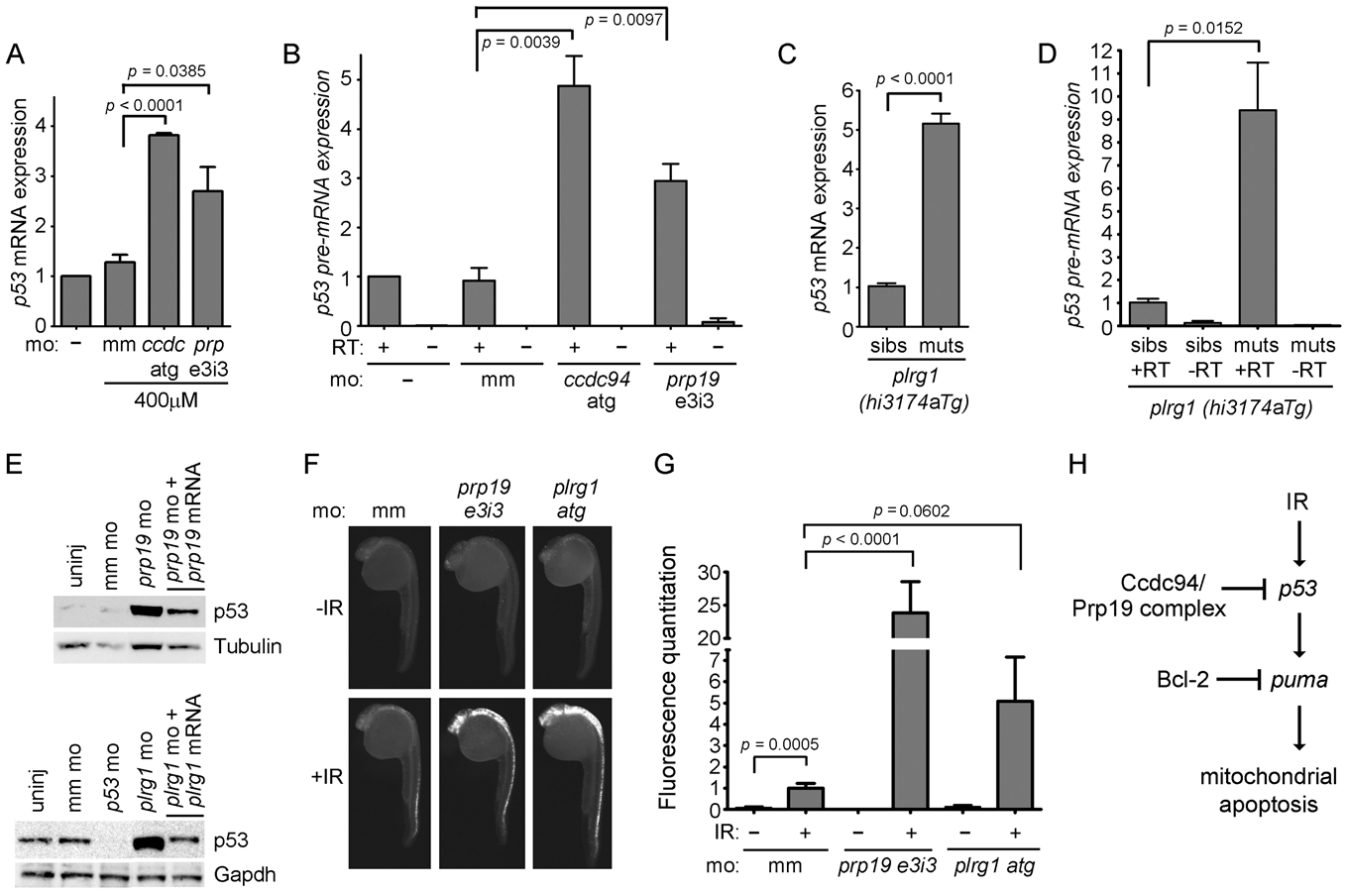
Loss of *prp19* or *plrg1* phenocopies, loss of *ccdc94*. (A) Wild-type embryos were injected with the indicated morpholinos. RNA was harvested at 30 hpf, reverse transcribed using oligo-dT primers and analyzed for the expression of *p53* mRNA by qPCR. Expression of the *gapdh* gene was also analyzed to normalize *p53* mRNA levels. All values were then compared to the average value from uninjected embryos, which was adjusted to one. (B) RNA from (A) was reverse transcribed with random hexamers, and intron 9 of the *p53* gene was analyzed by qPCR to determine levels of *p53* pre-mRNA. Expression of *28S* RNA was also analyzed to normalize *p53* pre-mRNA levels. All values were then compared to the average value from uninjected embryos, which was adjusted to one. (C, D) Siblings and mutants from the *plrg1(hi3174aTg)* line were distinguished by morphology at 30 hpf and collected for analysis. RNA was harvested and analyzed by qPCR as in (A, B). For panels (A–D), error bars represent the standard error of the mean from at least three independent experiments. (E) Wild-type embryos were injected with the indicated morpholinos and analyzed at 30 hpf for p53 protein similar to Figure 3E. (F) Wild-type embryos were injected with the indicated morpholinos, irradiated (or not) at 24 hpf with 8 Gy and analyzed three hours later by whole-mount immunofluorescence to detect activated Caspase-3. Three independent experiments showed that knockdown of *prp19* or *plrg1* radiosensitized the embryos. (G) Activated-Caspase-3-specific immunofluorescence from (F) was quantified as in Figure 1D. (H) Genetic diagram showing that the Ccdc94 and core members of the Prp19 complex inhibit the transcription of *p53*, and therefore p53-mediated induction of *puma* expression, and normally restrict IR-induced mitochondrial apoptosis, upstream of Bcl-2. For all relevant panels in this figure, morpholinos were injected at the same concentrations as described in Figure 4B-4D. RT; reverse transcriptase, mm; mismatch, *ccdc; ccdc94, prp; prp19*. See also Figure S8.

To determine whether the radioprotective function of Ccdc94 is derived from its interaction with the Prp19 complex, we questioned whether loss of *prp19* or *plrg1* could phenocopy the *rs7* mutation. We injected the *prp19* morpholino into wild-type embryos and analyzed expression of *p53* mRNA by qPCR. To ensure that we were analyzing full-length *p53* mRNA and not a truncated isoform that has been shown to be non-specifically induced by morpholino off-target effects [25], we used primers that were designed to amplify across exons 1 and 2 of the *p53* mRNA. The *ccdc94* morpholino and a mismatch morpholino were injected as positive and negative controls for induction of *p53* mRNA, respectively. The *prp19* morpholino independently caused a significant increase in *p53* mRNA and pre-mRNA expression, similar to knockdown of *ccdc94* (Figure 5A–5B). Of note, upregulation of *p53* transcripts in the *ccdc94* and *prp19* morphants was reduced compared to that observed in the *rs7* and *plrg1* mutants, likely due to incomplete knockdown by the morpholinos.

We next took advantage of a *plrg1* mutant zebrafish line [*plrg1(hi3174aTg*), [39]]. This line contains a retroviral insertion in intron 1 of the *plrg1* gene that leads to severely reduced mRNA levels (Figure S8A). Embryos that are homozygous for this retroviral insertion exhibit major developmental cell death in the central nervous system and usually die by the end of day two [39]. Injection of wild-type *plrg1* mRNA completely rescued this developmental phenotype at 24 hpf (Figure S8B). We analyzed *p53* mRNA and pre-mRNA levels in the *plrg1* mutants and found that, similar to the *rs7* mutants, they express abnormally high levels of *p53* mRNA and pre-mRNA (Figure 5C–5D).

We reasoned that the increase in *p53* expression in response to knockdown or loss of *prp19* and *plrg1*, respectively, should lead to an increase in IR-induced apoptosis, similar to knockdown or loss of *ccdc94* (Figure 2E–2F and Figure 1C, respectively). Since the *plrg1(hi3174aTg*) mutants have severe neurodegeneration that interfered with our ability to evaluate irradiated embryos for an increase in apoptosis, we elected to use the *plrg1* morpholino [38] to titrate *plrg1* expression levels. We first analyzed whether knockdown of *prp19* and *plrg1* would phenocopy the *rs7*-mediated increase in p53 protein expression seen in Figure 3E. Figure 5E shows that knockdown with either the *prp19 e3i3* or *plrg1 atg* morpholinos caused an increase in p53 expression that was specifically rescued by overexpression of the respective wild-type mRNAs. Knockdown of either *prp19* or *plrg1* also sensitized the embryos to IR-induced apoptosis (Figure 5F–5G). Together these results suggest that Ccdc94 is a component of the Prp19 complex which functions to protect proliferating embryonic neural cells from genotoxic stresses such as IR by modulating the levels of *p53* mRNA expression (Figure 5H).

## Discussion

We have previously shown that zebrafish embryonic neural tissue is an excellent model system to dissect the DSB-DDR pathway since it faithfully recapitulates many of the complex molecular signaling events elucidated in mammalian systems [4], [12]. With the notion that conserved embryonic pathways are exploited by cancer cells to promote survival, we embarked on a unique approach to use an unbiased genetic screen in zebrafish to identify novel radioprotective genes in the DSB-DDR pathway. The goal of these studies is to better understand the role of the DSB-DDR pathway in development, knowledge that can be ultimately translated to improved therapies for cancer treatment. Our efforts led us to identify a novel radioprotective gene called *ccdc94*. The Ccdc94 *S. cerevisiae* ortholog (named Yju2) has previously been shown to interact with the Prp19 complex [14], [30], [32] and is required for the first catalytic step in pre-mRNA splicing in yeast [14]. Our experiments confirm that the Ccdc94/Prp19 complex interaction is conserved in vertebrate cells; however, we have identified a new function for this complex in repressing *p53* mRNA expression during development.

The Prp19 complex has a well-established role in pre-mRNA splicing in yeast [15]–[16], but whether the complex has an equivalent role in vertebrates is not clear [17]. Our studies show that the Prp19 complex normally represses *p53* mRNA expression in neural cells to promote cell survival. There is a large body of evidence documenting the regulation of p53 activity by post-translational mechanisms [40] but a relative dearth of studies addressing potential mechanisms by which *p53* is regulated at the transcriptional level despite the fact that potent transcriptional up-regulation could conceivably overcome post-translational mechanisms that restrict p53 activity. Indeed, we found that the 9-fold up-regulation of *p53* mRNA transcripts in *rs7* mutants translates to increased p53 activity. To date, there is no evidence to suggest that the Prp19 complex directly regulates transcription. An analysis of down-regulated genes in the *rs7* mutants could yield novel, direct mechanisms by which the *p53* gene is transcriptionally regulated.

The Prp19 complex has also been linked to DNA repair [17]. Indeed, the *prp19* gene was originally identified in *S. cerevisiae* in a screen for radiosensitizing mutations [33]. The *prp19* mutant identified in this screen, initially called *xs9*, was subsequently renamed *pso4-1* since it was shown to have much greater sensitivity to the DNA interstrand cross-linking (ICL) agent PAVA (psoralen plus UVA light therapy) than to IR [34]. Further analysis of this thermoconditional mutant revealed that Prp19 functions in repair of DNA ICLs in a pathway that appears to be genetically separable from its role in pre-mRNA splicing [33]–[34], [36]. Studies of human PRP19 confirmed the role of the PRP19 complex in ICL repair [37] and also suggested a role for PRP19 in the repair of DNA DSBs [35]. Therefore, the increase in *p53* expression resulting from loss of Prp19 complex gene expression could ultimately stem from a lack of DNA repair.

The neural tissue is one of the most radiosensitive tissues in the early developing zebrafish embryo. One likely explanation arises from the supposition that developing neurons are already poised to undergo apoptosis if they fail to receive neurotrophic survival factors from their synaptic targets [41]. As such, readiness to undergo DNA-damage-induced apoptosis is a feature that embryonic neural tissue shares with many cancers [42]. The neural tissue is also one of the most highly proliferative tissues in the developing embryo (our unpublished observations), another feature in common with cancer [43]. By exploiting the similarities between embryonic neural tissue and cancer cells, we have been able to use an unbiased genetic approach to identify novel and conserved genes involved in the DSB-DDR that represent potentially important targets in both neurodegenerative disease and radio-resistant cancers.

Sensitivity to IR-induced apoptosis by the *rs7* mutation appears to be restricted to neural tissue and possibly primitive hematopoietic tissue in the ICM (Figure 1C). These areas are consistent with disproportionately high levels of *ccdc94* mRNA expression during zebrafish embryonic development (http://zfin.org/cgi-bin/webdriver?MIval=aa-pubview2.apg&OID=ZDB-PUB-040907-1 and data not shown) as well as regions of high *p53* mRNA expression (Figure 3B) in *rs7* mutants. In general, tissues other than neural or ICM have modest expression of *ccdc94* by comparison suggesting that Ccdc94-independent mechanisms evolved to regulate *p53* expression in these other tissues. However, *CCDC94* appears to be ubiquitously expressed in tissues of adult humans (http://www.ncbi.nlm.nih.gov/UniGene/clust.cgi?ORG=Hs&CID=21811) suggesting that it may have a broader role in the regulation of *p53* expression beyond development.

In conclusion, we have identified and analyzed the role of a novel radioprotective gene, *ccdc94*, in the first genetic screen in a vertebrate system designed to identify radiosensitizing mutations *in vivo*. We found that in vertebrate cells Ccdc94 interacts with core members of the Prp19 complex both biochemically and genetically and that protection from IR-induced apoptosis by this complex is mediated by inhibition of *p53* expression. Upregulation of *p53* expression could potentially overcome mechanisms that evolve during cancer progression to restrict its protein activity. Future experiments will determine whether CCDC94 and PRP19 complex components will be useful targets for sensitizing *p53* wild-type cancer cells to IR therapy.

## Materials and Methods

### Ethics statement

All experiments involving zebrafish conformed to the regulatory standards and guidelines of the Dana-Farber Cancer Institute and University of Utah Institutional Animal Care and Use Committee.

### Zebrafish lines

Zebrafish were maintained, mutagenized and bred as described [44]. Wild-type embryos and the *rs7* mutant line were derived from the AB strain. The *rs7* mutant line was outcrossed to wild-type AB fish seven times and all phenotypes described herein are representative of crosses between at least seventh generation *rs7* heterozygous fish. The *plrg1(hi3174aTg*) line was obtained from the Zebrafish International Resource Center (http://zebrafish.org/zirc/home/guide.php). We have previously described the *p53^e7/e7^* zebrafish line that carries a homozygous M214K mutation in the *p53* coding sequence [26].

### Irradiator usage

IR was administered with either a Cs-137 gamma irradiator (Gammacell 1000) or an X-ray irradiator (RadSource RS2000). Irradiators were completely interchangeable such that 8 Gy of X-rays gave rise to identical embryonic phenotypes described in this study as 8 Gy of gamma rays.

### Zebrafish microinjections

Zebrafish one-cell stage embryos were injected with 500 picoliters of mRNA or the indicated concentration of morpholino. In every experiment, total RNA or morpholino concentrations were kept constant through the addition of *egfp* mRNA or a mismatch morpholino, respectively. Morpholinos were designed and created by GeneTools, and sequences are listed in Text S1. For mRNA microinjection, zebrafish cDNAs were sub-cloned into pCS2+, and mRNA was made by 1) linearization of each construct with NotI, 2) SP6 Message Machine kit (Ambion, AM1340) and 3) purification for microinjection with NucAway Spin Columns (Ambion, AM10070). For morpholino rescue experiments, the *plrg1* coding sequence was mutated to prevent direct binding of *plrg1* mRNA to the translation-blocking *plrg1 atg* morpholino while creating only silent changes in regard to Plrg1 amino acid sequence (relevant primers are listed in Text S1).

### Whole-mount *in situ* hybridization

For *in situ* hybridization, zebrafish cDNAs were sub-cloned into the pGEM-T-Easy (Promega). The *pGEM-puma* vector and generation of antisense probe was described previously [46]. The full-length zebrafish *tp53* coding sequence was amplified with primers based on the published GenBank sequence (NM_131327 and Text S1). Full-length antisense and sense *p53* RNA was generated by digesting with Apa1 and EcoRI, respectively, and transcribed in vitro with Sp6 and T3 polymerase, respectively. Embryos were dechorionated, staged, and fixed overnight in 4% paraformaldehyde at 4°C. Fixed embryos were washed in 1× PBST (1× PBS plus 0.1% Tween-20) at room temperature (RT, 3×10 minutes) and incubated in 100% methanol at −20°C for a minimum of two hours. Embryos were rehydrated in 1× PBST (3×10 minute washes at RT) and incubated in Hyb-minus (50% formamide, 5× SSC, and 0.1% Tween-20) for one hour at 68°C, then transferred to Hyb-plus (Hyb-minus, 5 mg/ml torula RNA type VI, 50 ug/ml heparin) for three hours at 68°C. RNA probe was added to embryos in Hyb-plus at 1 ng/uL and incubated overnight at 68°C. Embryos were then subjected to 20-minute washes at 68°C in the following order: twice with 2× SSCT-formamide (2× SSC, 0.1% Tween-20, 50% formamide), once with 2× SSCT, twice with 0.2× SSCT. At RT, embryos were then washed (3×10 minutes) with MABT (100 mM maleic acid, 150 mM sodium chloride, 100 mM Tris pH 9.5, 0.1% Tween-20), then incubated in block [MABT, 2% Blocking Reagent (Roche #11096176001), 1% fetal bovine serum] at RT for one hour. Embryos were incubated overnight in 1∶5000 anti-digoxigenin-fluorescein Fab fragments (Roche #11207741910) in block at 4°C. Embryos were then washed in 1× MABT (3×10 minutes at RT) and 0.1 M Tris pH 9.5 (3×10 minutes at RT) and stained with Vector BCIP/NBT alkaline phosphatase (Roche #11697471001). Upon completion of staining, embryos were washed in 1× PBST (3×10 minutes at RT) to stop the reaction. Embryos were imaged in either 80% glycerol or 3% methylcellulose (to allow for subsequent genotyping).

### Whole-mount immunofluorescence and quantitation

Whole-mount activated Caspase-3 immunofluorescence was performed and quantified as described previously [12]. Quantitation was performed after immunofluorescence experiments by removing embryo tails at the level of the yolk. Tails were laid flat on a petri dish in 80% glycerol and activated Caspase-3 staining was documented by fluorescence microscopy using a Nikon Digital Sight DS-2MBWc black and white camera. All fluorescent pictures were taken at exactly the same exposure, gain, and magnification. Pictures were then cropped in Adobe Photoshop to include the same size region (using the ruler function on Photoshop) of the spinal cord using the end of the yolk extension as a reference point for all measurements, and to exclude any fluorescence arising outside of the spinal cord. Quantitation of fluorescence was performed with Volocity software by using the “Find Objects Using Intensity” option. The same exact parameters for eliminating the inclusion (and therefore quantitation) of background fluorescence were applied to all pictures equally. Six to twelve embryos from each group were included for all quantifications. GraphPad Prism software was used to plot the data, and error bars represent the standard error of averaged data from the embryos in a single experiment. Statistical analyses were performed using GraphPad Prism software using an unpaired student's T test. Representative quantitations from at least three experiments are shown for all data. Quantification represents measurements of fluorescence intensity which is directly related to Caspase-3 activity. However, fluorescence intensity is likely to fluctuate within cells. Therefore, changes in fluorescence intensity likely represent both increasing apoptotic cell number as well as increasing Caspase-3 activity within cells.

### RNA–Seq analysis

The transcriptome of 30 hpf wild-type, *rs7* siblings and *rs7* mutants was analyzed by Illumina RNA–Seq analysis. Total RNA samples were prepared for sequencing using an Illumina mRNA Seq Sample Prep Kit and were sequenced using standard protocols on an Illumina Genome Analyzer IIx. Paired 36 base pair reads were obtained from the ends of each sequenced fragment. Reads were aligned to the Danio rerio Zv8 genome build (release date December 2008, obtained from UCSC Genome Bioinformatics, http://genome.ucsc.edu) with the SOAPAligner software (release 2.19, http://soap.genomics.org.cn/index.html). The SOAPAligner was run to allow an insert range of 100 to 140 bp, as the mean library insert size was 120 bp. Sequencing and alignment of the samples yielded the following in terms of sample, read pairs, aligned reads, and percent aligned: 1) *rs7* muts, 19,034,491, 18,358,098, 96.4%, 2) *rs7* sibs, 20,036,828, 19,206,760, 95.9%, 3) wild-type, 16,604,110, 12,630,153, 76.1%. RNA–Seq data from the *rs7* mutant, sibling, and wild-type samples was analyzed for differential expression and differential splicing using the DefinedRegionScanSeqs method of the USeq software [45]. Transcript coordinates for this analysis were collected from the UCSC Genome Browser database [46].

### Quantitative real-time PCR

RNA was isolated from embryos (10–25 embryos/sample) using the Qiagen RNeasy kit (74104). One microgram of purified RNA was used to generate cDNA using the Invitrogen Thermoscript RT-PCR kit (11146-024). For mRNA analysis, RNA was reverse transcribed using oligo-dT primers. For pre-mRNA analysis, RNA was reverse transcribed with random hexamers. cDNA was diluted 1∶20 in nuclease-free water and three technical replicates were analyzed using the LightCycler 480 Probes Master PCR mix (4707494001) and the Roche 480 Light Cycler or Eppendorf Realplex. Primers were designed by Roche to be used with their Universal Probe Library and are listed in Text S1. Primers were designed to cross exons 1 and 2 of the *p53* gene to ensure specific amplification of full-length *p53* mRNA, and expression of the *gapdh* gene was analyzed to normalize *p53* mRNA levels. Introns 4 and 9 of the *p53* gene were analyzed by qPCR to determine levels of *p53* pre-mRNA, and expression of *28S* RNA (which does not undergo splicing) was analyzed to normalize *p53* pre-mRNA levels. All data was averaged from at least three independent experiments. GraphPad Prism software was used to plot the data, and error bars represent the standard error of averaged data. Statistical analyses were performed using GraphPad Prism software using an unpaired student's T test.

### Tandem affinity purification/mass spectrometry analysis

*CCDC94* cDNA was cloned into the pGlue vector encoding a dual-affinity tag containing streptavidin-binding protein, calmodulin-binding protein, and the hemagglutinin epitope. Lines of 293T cells expressing low levels of the tagged-bait fusion proteins were generated, detergent-solubilized, subjected to two rounds of affinity purification, trypsinized, and analyzed by liquid chromatography–tandem mass spectrometry. Tryptic peptides were separated by reverse phase nano-HPLC using a nanoAquity UPLC system (Waters Inc). Peptides were first trapped in a 2 cm trapping column (75 µm ID, C18 beads of 2.5 mm particle size, 200 Å pore size) and then separated on a self-packed 25 cm column (75 µm ID, C18 beads of 2.5 mm particle size, 100 Å pore size) at room temperature. The flow rate was 350 nl/min over a gradient of 5% buffer B (0.1% formic acid in acetonitrile) to 40% buffer B in 200 minutes. The identity of the eluted peptides was determined with a Velos-Orbitrap mass spectrometer (Thermo-Scientific). Specifically, following a FT full scan, MS^2^ spectral data were acquired by collision induced dissociation (CID) on the 9 most intense ions from the full scan, taking into account dynamic exclusion. The polysiloxane lock mass of 445.120030 was used throughout. All raw data were converted to mzXML format before a semi-tryptic search of the resultant spectra using Sequest and the Transproteomic Pipeline (TPP) on a Sorcerer 2.0 platform (Sage N Research, Milpitas, CA).

### Western analysis

Approximately 100 30-hpf dechorionated embryos were rinsed three times at RT with 1× PBS. Ice-cold PBS was added, and embryos were incubated on ice for 5 minutes. Embryos were then de-yolked by pipetting 40 times through a thin bore plastic bulb pipet (Samco 235) in ice-cold PBS. Tubes were returned to ice for two minutes to let embryo bodies sink to the bottom of the tube. Supernatant was removed and embryo bodies were washed three times in ice-cold PBS, pelleted by centrifugation (1 second at top speed), and lysed with RIPA buffer (1% Nonide P-40, 0.5% sodium deoxycholate, 0.1% sodium dodecyl sulfate) containing 1% protease inhibitors (Sigma P8340) and 1% benzonase (Novagen 70746-3). BCA protein assay kit (Thermo Scientific 23227) was used to determine protein concentration. Ten micrograms of protein was loaded onto a denaturing gel (Novex NP0301BOX) and transferred to pre-hydrated PVDF membrane (GE PV4HYA0010). Membrane was blocked in 10% non-fat milk diluted in tris-buffered saline plus 1% tween-20 (TBST) for anti-p53 antibody. Zebrafish p53 antibody was kindly provided by Dr. Lane at A*STAR, Singapore and used at 1∶30 in 10% milk with overnight incubation at 4°C. The blot was washed (4×5 minutes each) in 1× TBST. Anti-mouse-horseradish peroxidase secondary antibody (Cell Signaling 7076S) was used at 1∶5000 in the same block for primary antibody. The blot was washed (4×5 minutes each) in 1× TBST before chemiluminescent horseradish peroxidase substrate (Millipore WBKLS0500) and film (Thermo Scientific 34090) were used to detect signal. Blot was stripped for 45 minutes rotating at 60°C (62.5 mM Tris, 2% sodium dodecyl sulfate, 100 mM 2-mercaptoethanol) and washed (8×15 minutes at RT) in TBST. The blot was then re-blocked in 3% bovine serum albumin (Amresco 0332) in TBST and probed using anti-GAPDH antibody (Abcam 9484) at 1∶2000 or anti-α-Tubulin antibody (Sigma T9026) at 1∶10,000. The mouse secondary antibody and detection was performed as outlined above. Developed films were electronically scanned at 600 dots per inch and quantified using ImageJ software.

## Supporting Information

Figure S1 Schematic of genetic screen to identify recessive radiosensitizing mutations. Wild-type AB strain male zebrafish were treated with ENU giving rise to approximately 192 gene-inactivating mutations per sperm. ENU-mutagenized males were crossed with wild-type AB strain female fish to yield the F1 generation. Each fish in the F1 generation carried 192 unique mutations and were incrossed to create F2 families carrying 384 mutations per family. Up to eight random incrosses were performed within each F2 family, and the F3 clutches were subsequently analyzed for radiosensitizing phenotypes as described in the figure.
(TIF)

Figure S2 Alignment of zebrafish, mouse and human CCDC94 protein sequences. Protein sequences from zebrafish, mouse and human CCDC94 were aligned with the Jotun Hein algorithm using the default parameters in Lasergene MegAlign software. Zebrafish Ccdc94 is 67% identical to human CCDC94 and 69% identical to mouse CCDC94. An asterisk denotes R125, the residue that is mutated to a stop codon in *rs7* mutants.
(TIF)

Figure S3 The *rs7* mutation causes severe neurodegeneration that culminates in embryonic lethality. Wild-type embryos (derived from wild-type parents) and *rs7* mutants were analyzed by brightfield microscopy on day 2 and day 3 of development. The *rs7* mutation causes massive accumulation of cell death in the brain and spinal cord likely contributing to a small-head and “curly-up” tail phenotype. *Rs7* mutant embryos usually fail to survive past day three.
(TIF)

Figure S4 Loss of *ccdc94* compromises the anti-apoptotic function of Bcl-2. Wild-type (wt, derived from crossing wild-type parents) or *rs7* mutant embryos (derived from crossing *rs7* heterozygous parents) were injected at the one-cell stage of development with the indicated amounts of mRNA encoding *zbcl-2, zbcl-xL,* or *egfp* as a control (cntl). Embryos were then irradiated at 24 hpf with 8 Gy IR and analyzed three hours later by immunofluorescence to detect activated Caspase-3. Mutants were genotyped as in Figure 2C–2D. Representative data from three independent experiments is shown.
(TIF)

Figure S5 The *rs7* mutation does not appear to cause a general activation of the DSB-DDR pathway. (A) Wild-type embryos were exposed (or not) to 8 Gy IR at 27 hpf and harvested for analysis three hours later. Quantitative PCR to analyze *p53* mRNA expression was performed as in Figure 3A. (B) *Rs7* siblings and mutants in the p53*^e7/e7^* background [26] were analyzed at 30 hpf for expression of the indicated genes by qPCR similar to Figure 3A. For both (A) and (B), *gapdh* mRNA expression was measured to normalize gene expression levels. All data was then compared to *rs7* sibling data, which was adjusted to a value of one. None of the values in (B) are significantly different from each other.
(TIF)

Figure S6 p53-mediated *puma* expression causes neurodegeneration in *rs7* mutants. (A) *rs7* sibling or mutant embryos in the *p53* wild-type or mutant background were collected, and RNA was harvested at 30 hpf, reverse transcribed and analyzed for the expression of *puma* mRNA by qPCR. *Rs7* mutants and siblings were distinguished by morphology since loss of p53 does not inhibit the *rs7*-mediated “curly-up” tail phenotype. (B) Pictures were taken of 48 hpf embryos from (A). Arrows point to neurodegeneration in *rs7* mutants that is substantially rescued by loss of wild-type *p53*. For panel (A), error bars represent the standard error from three independent experiments.
(TIF)

Figure S7 The *prp19* morpholino inhibits proper splicing of the *prp19* pre-mRNA transcript. (A) Schematic of the zebrafish *prp19* gene with exons shown as boxes and introns shown as lines between boxes. The *e3i3* morpholino targets the splice donor site at the border of exon 3 and intron 3. In (B), F1 and R2 primer-based PCR will amplify from exon 2 to intron 3. Properly spliced mRNA should not give rise to PCR product. Inclusion of intron 3 should give rise to a 245-basepair product. (B) RNA from embryos injected with mismatch morpholino controls or *prp19 e3i3* morpholino was harvested at 24 hpf, reverse transcribed with oligo-dT primers, and analyzed by PCR using primers shown in (A). Bands were excised from the gel, cloned into pGEM-T-Easy, and sequenced. Lower-case letters point to bands on the gel which correspond to regions of the *prp19* gene shown below. When embryos were injected with the *e3i3* morpholino, F1/R2 primers gave rise to a band representing inclusion of intron 3 (b). This should cause premature termination the Prp19 protein since a stop codon is present in the middle of the intron. Unexpectedly, all samples gave rise to a 330-basepair band (a) which is likely derived from the presence of contaminating genomic DNA but does not change interpretation of the data.
(TIF)

Figure S8 Loss of *plrg1* mRNA expression in the *plrg1(hi3174aTg)* line leads to severe developmental abnormalities characterized by excessive cell death. (A) Siblings and mutants were collected from the *plrg1(hi3174aTg)* line based on morphology. RNA was harvested from each group at 30 hpf, reverse transcribed and analyzed by qPCR to determine levels of *plrg1* mRNA. Error bars represent the standard error from three independent experiments. (B) *plrg1(hi3174aTg)* heterozygous fish were incrossed, and the progeny were either left uninjected or injected with 100 ng/uL of the indicated mRNAs. Right panels show higher magnification of embryos from adjacent left panels. Overexpression of *plrg1* rescues the developmental abnormalities in the *plrg1(hi3174aTg)* mutants.
(TIF)

Text S1 Supplemental Experimental Procedures. (A) Primers used for subcloning are listed and include the name of the target gene as well as restriction sites used for directional cloning into the pCS2+ expression plasmid. (B) The sequences of morpholinos used in this study are listed. The *plrg1* morpholino was previously described [38]. (C) Primers used to examine specificity of the *prp19 e3i3* morpholino are listed. See also Figure S7. (D) Primers used to sub-clone *zp53* into pGEM-T-Easy for use in *p53 in situ* hybridization experiments are shown. See also Figure 3B. (E) Primer/probe sets used for qPCR analysis are grouped by presence or absence of shading.
(PDF)
