# Supplementary figures and images for: Ccdc94 Protects Cells from Ionizing Radiation by Inhibiting the Expression of p53
Source: PLoS Genet. 2012 Aug 30;8(8):e1002922. doi: 10.1371/journal.pgen.1002922 (PMC3431329; doi:10.1371/journal.pgen.1002922)

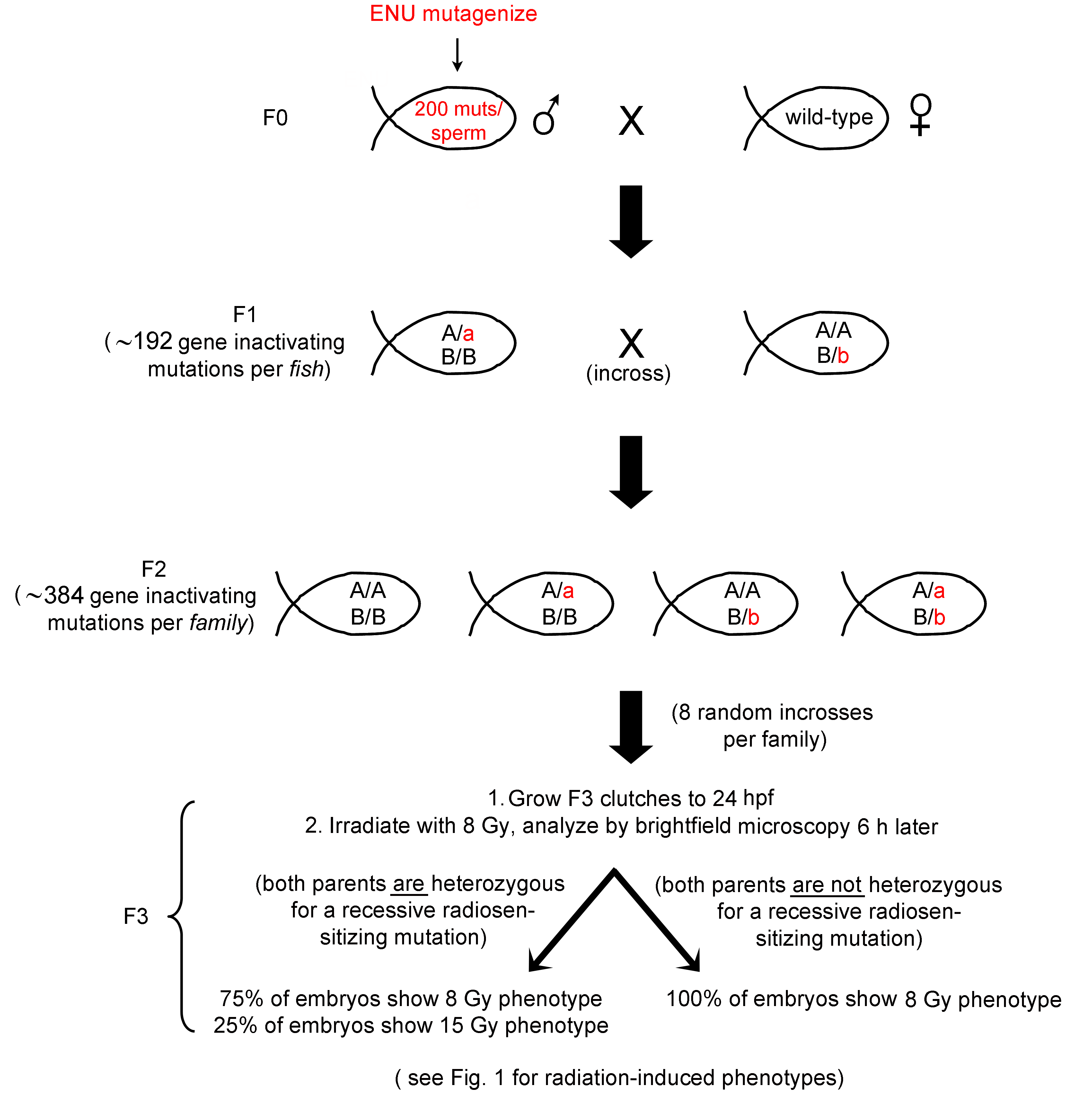

Supplement: Figure S1 — Schematic of genetic screen to identify recessive radiosensitizing mutations. Wild-type AB strain male zebrafish were treated with ENU giving rise to approximately 192 gene-inactivating mutations per sperm. ENU-mutagenized males were crossed with wild-type AB strain female fish to yield the F1 generation. Each fish in the F1 generation carried 192 unique mutations and were incrossed to create F2 families carrying 384 mutations per family. Up to eight random incrosses were performed within each F2 family, and the F3 clutches were subsequently analyzed for radiosensitizing phenotypes as described in the figure. (TIF) [file pgen.1002922.s001.tif]

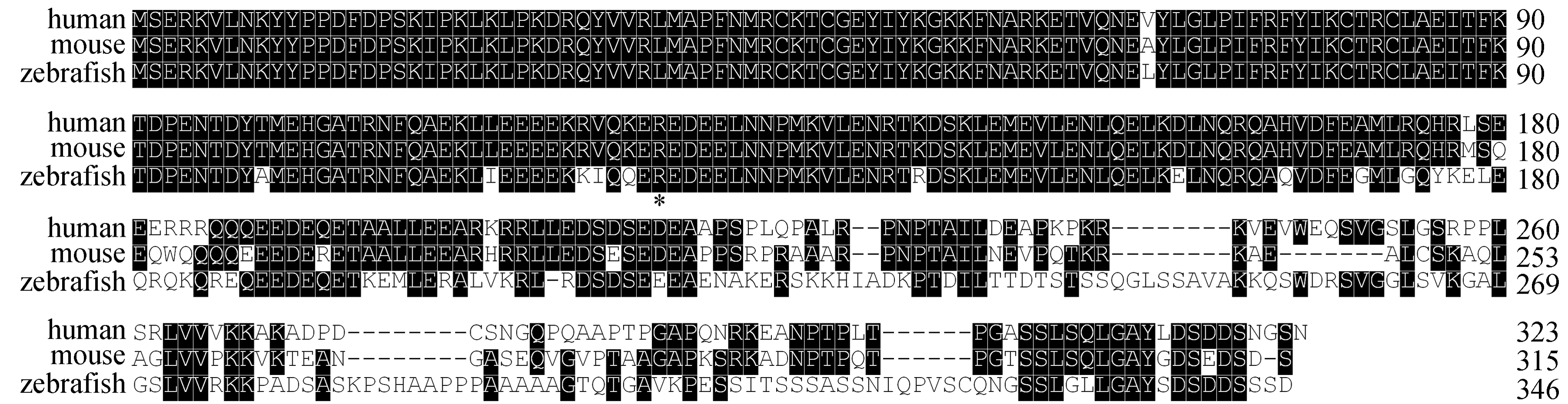

Supplement: Figure S2 — Alignment of zebrafish, mouse and human CCDC94 protein sequences. Protein sequences from zebrafish, mouse and human CCDC94 were aligned with the Jotun Hein algorithm using the default parameters in Lasergene MegAlign software. Zebrafish Ccdc94 is 67% identical to human CCDC94 and 69% identical to mouse CCDC94. An asterisk denotes R125, the residue that is mutated to a stop codon in rs7 mutants. (TIF) [file pgen.1002922.s002.tif]

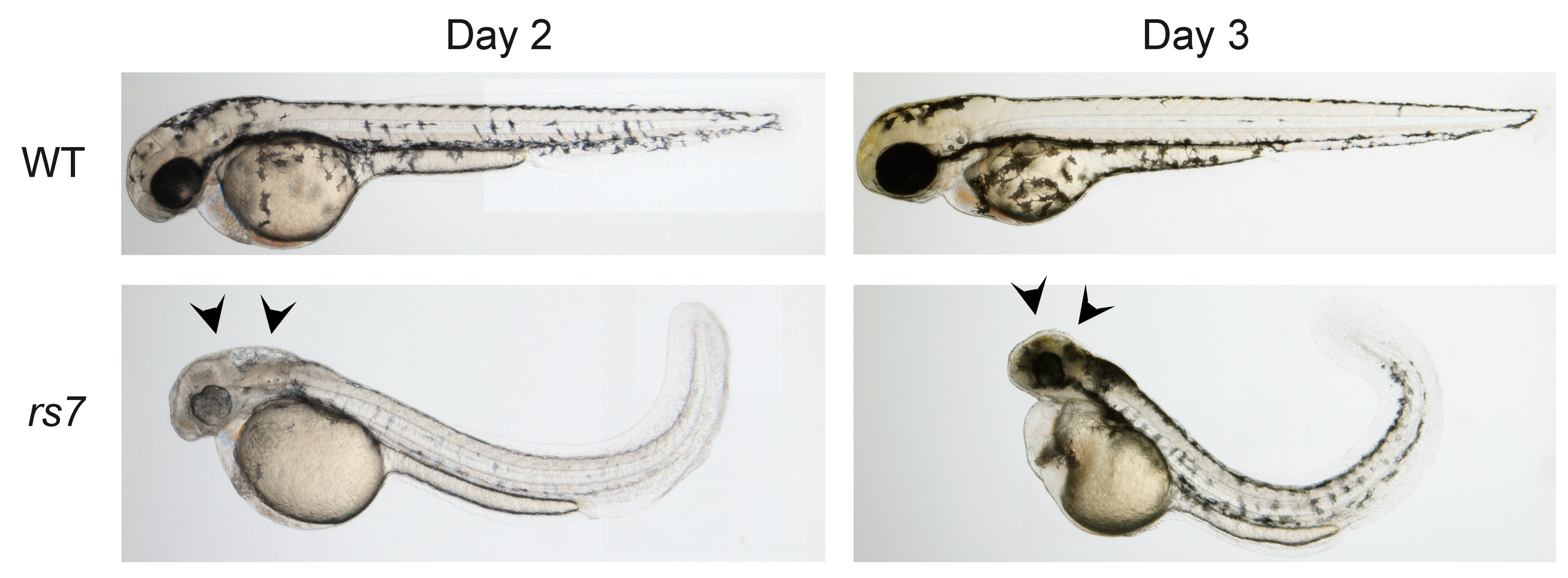

Supplement: Figure S3 — The rs7 mutation causes severe neurodegeneration that culminates in embryonic lethality. Wild-type embryos (derived from wild-type parents) and rs7 mutants were analyzed by brightfield microscopy on day 2 and day 3 of development. The rs7 mutation causes massive accumulation of cell death in the brain and spinal cord likely contributing to a small-head and “curly-up” tail phenotype. Rs7 mutant embryos usually fail to survive past day three. (TIF) [file pgen.1002922.s003.tif]

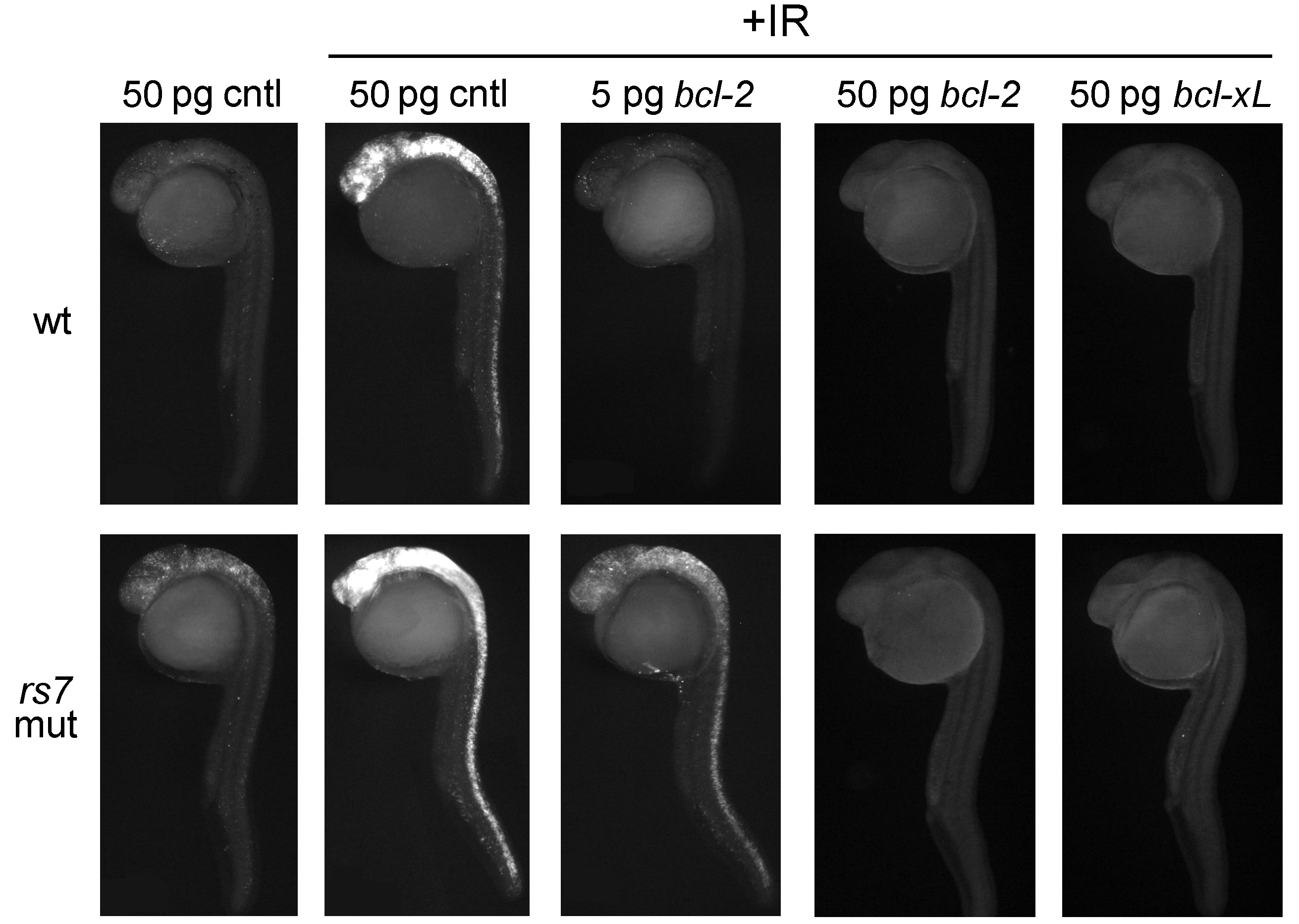

Supplement: Figure S4 — Loss of ccdc94 compromises the anti-apoptotic function of Bcl-2. Wild-type (wt, derived from crossing wild-type parents) or rs7 mutant embryos (derived from crossing rs7 heterozygous parents) were injected at the one-cell stage of development with the indicated amounts of mRNA encoding zbcl-2, zbcl-xL, or egfp as a control (cntl). Embryos were then irradiated at 24 hpf with 8 Gy IR and analyzed three hours later by immunofluorescence to detect activated Caspase-3. Mutants were genotyped as in Figure 2C–2D. Representative data from three independent experiments is shown. (TIF) [file pgen.1002922.s004.tif]

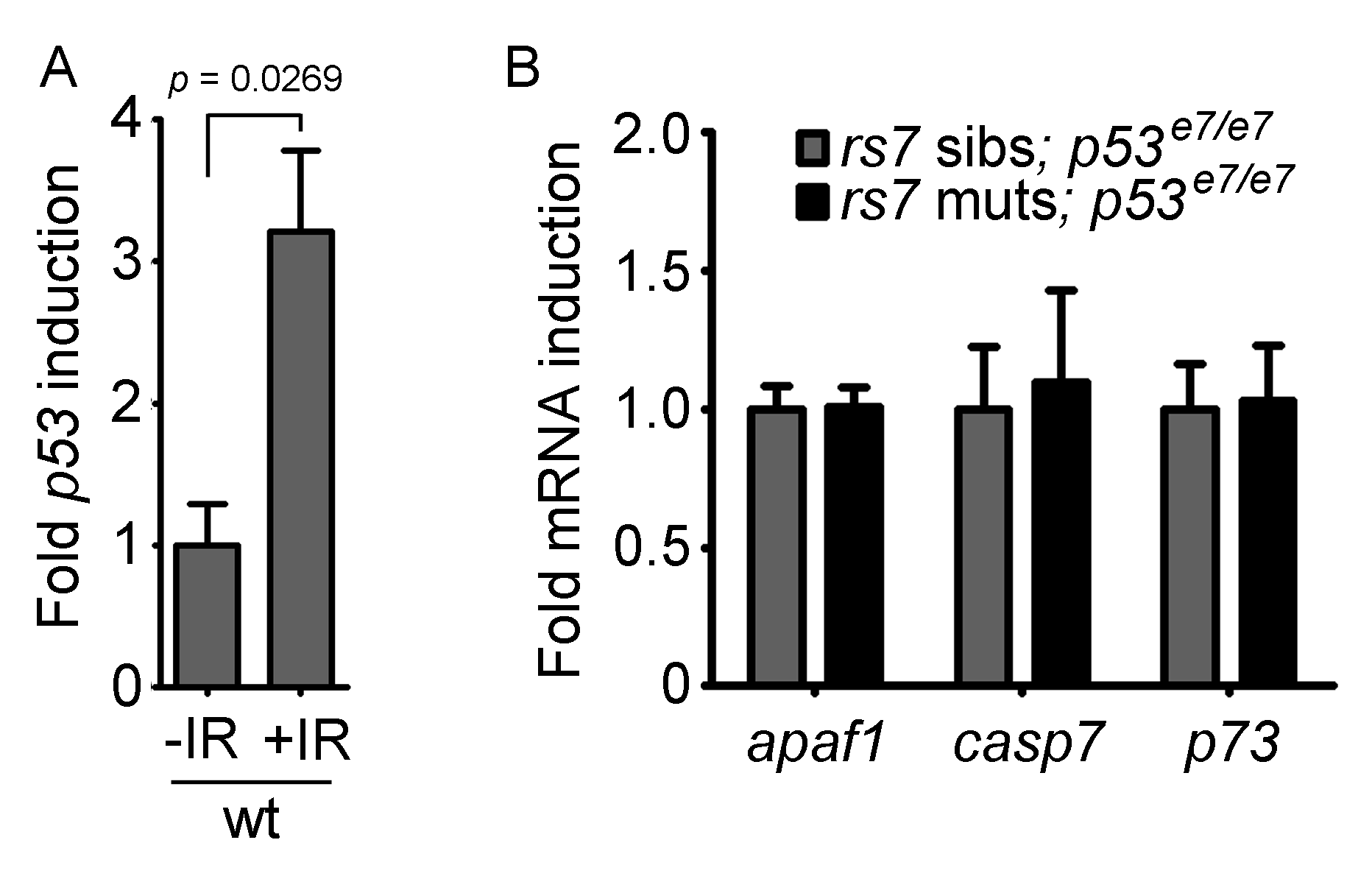

Supplement: Figure S5 — The rs7 mutation does not appear to cause a general activation of the DSB-DDR pathway. (A) Wild-type embryos were exposed (or not) to 8 Gy IR at 27 hpf and harvested for analysis three hours later. Quantitative PCR to analyze p53 mRNA expression was performed as in Figure 3A. (B) Rs7 siblings and mutants in the p53e7/e7 background [26] were analyzed at 30 hpf for expression of the indicated genes by qPCR similar to Figure 3A. For both (A) and (B), gapdh mRNA expression was measured to normalize gene expression levels. All data was then compared to rs7 sibling data, which was adjusted to a value of one. None of the values in (B) are significantly different from each other. (TIF) [file pgen.1002922.s005.tif]

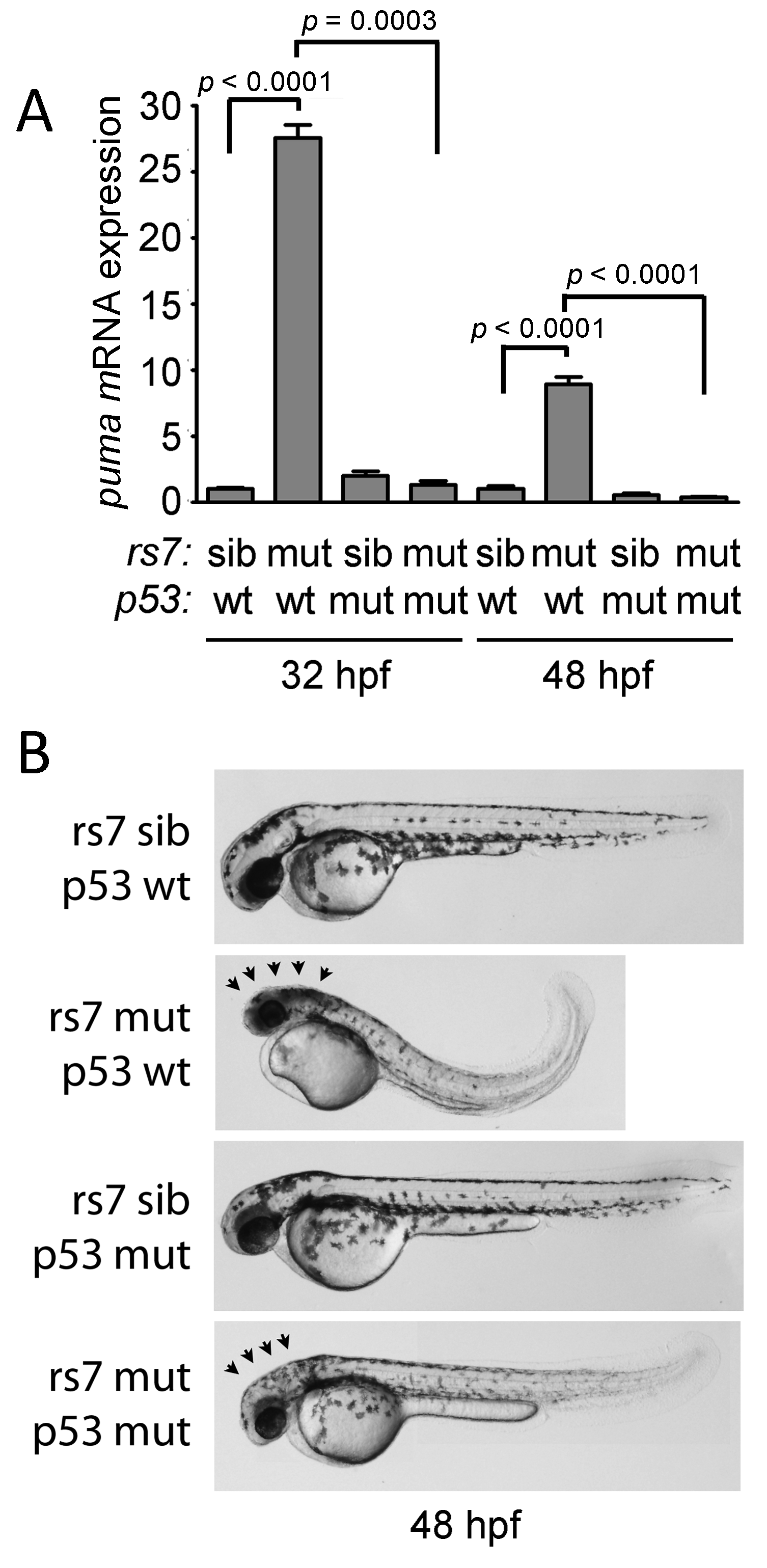

Supplement: Figure S6 — p53-mediated puma expression causes neurodegeneration in rs7 mutants. (A) rs7 sibling or mutant embryos in the p53 wild-type or mutant background were collected, and RNA was harvested at 30 hpf, reverse transcribed and analyzed for the expression of puma mRNA by qPCR. Rs7 mutants and siblings were distinguished by morphology since loss of p53 does not inhibit the rs7-mediated “curly-up” tail phenotype. (B) Pictures were taken of 48 hpf embryos from (A). Arrows point to neurodegeneration in rs7 mutants that is substantially rescued by loss of wild-type p53. For panel (A), error bars represent the standard error from three independent experiments. (TIF) [file pgen.1002922.s006.tif]

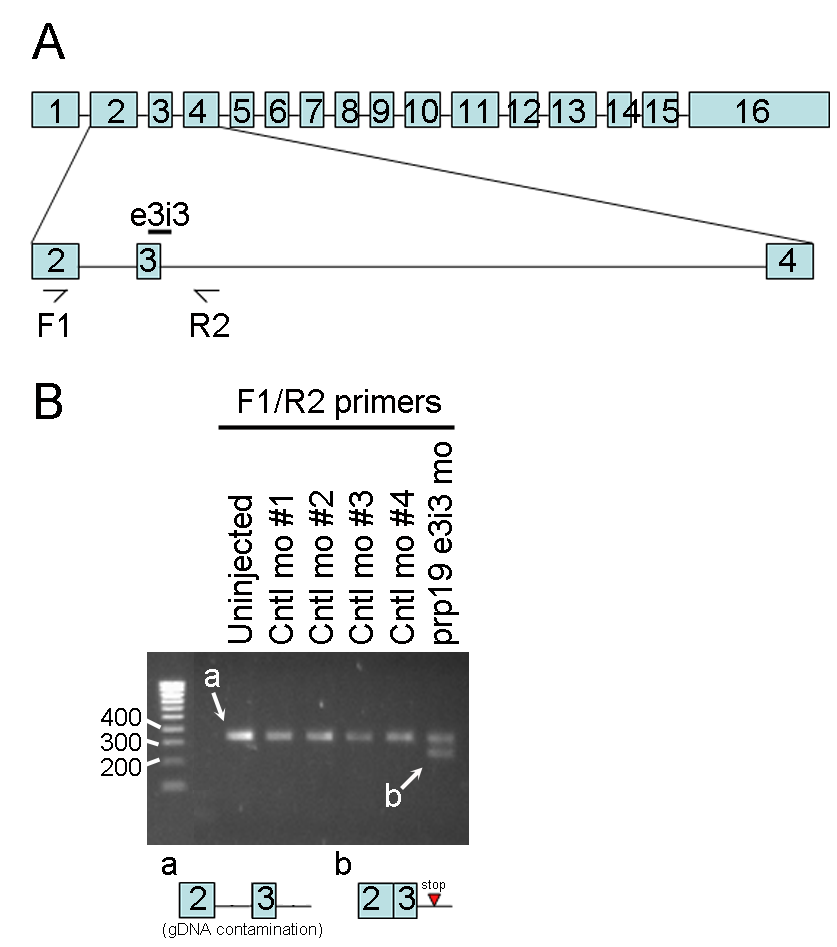

Supplement: Figure S7 — The prp19 morpholino inhibits proper splicing of the prp19 pre-mRNA transcript. (A) Schematic of the zebrafish prp19 gene with exons shown as boxes and introns shown as lines between boxes. The e3i3 morpholino targets the splice donor site at the border of exon 3 and intron 3. In (B), F1 and R2 primer-based PCR will amplify from exon 2 to intron 3. Properly spliced mRNA should not give rise to PCR product. Inclusion of intron 3 should give rise to a 245-basepair product. (B) RNA from embryos injected with mismatch morpholino controls or prp19 e3i3 morpholino was harvested at 24 hpf, reverse transcribed with oligo-dT primers, and analyzed by PCR using primers shown in (A). Bands were excised from the gel, cloned into pGEM-T-Easy, and sequenced. Lower-case letters point to bands on the gel which correspond to regions of the prp19 gene shown below. When embryos were injected with the e3i3 morpholino, F1/R2 primers gave rise to a band representing inclusion of intron 3 (b). This should cause premature termination the Prp19 protein since a stop codon is present in the middle of the intron. Unexpectedly, all samples gave rise to a 330-basepair band (a) which is likely derived from the presence of contaminating genomic DNA but does not change interpretation of the data. (TIF) [file pgen.1002922.s007.tif]

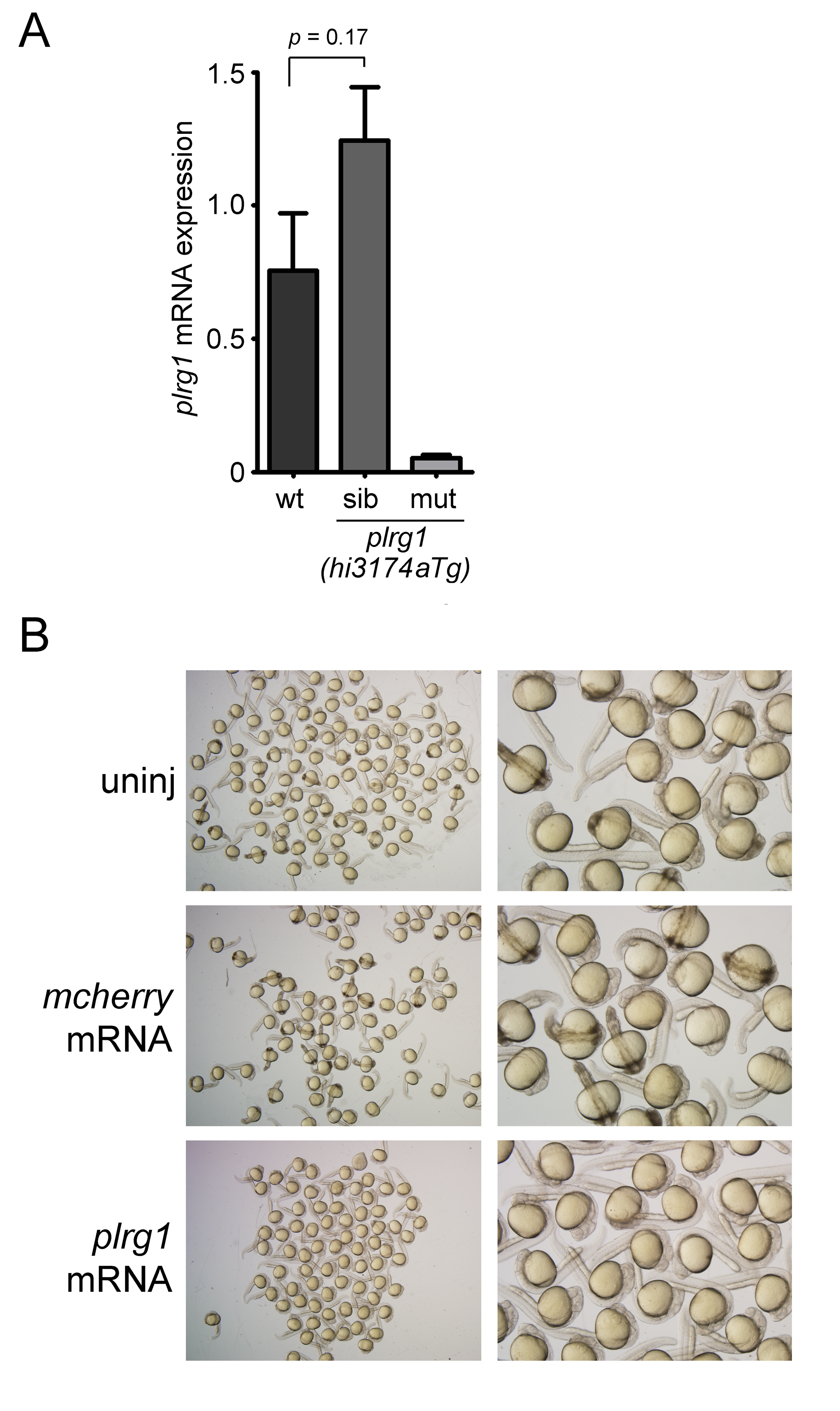

Supplement: Figure S8 — Loss of plrg1 mRNA expression in the plrg1(hi3174aTg) line leads to severe developmental abnormalities characterized by excessive cell death. (A) Siblings and mutants were collected from the plrg1(hi3174aTg) line based on morphology. RNA was harvested from each group at 30 hpf, reverse transcribed and analyzed by qPCR to determine levels of plrg1 mRNA. Error bars represent the standard error from three independent experiments. (B) plrg1(hi3174aTg) heterozygous fish were incrossed, and the progeny were either left uninjected or injected with 100 ng/uL of the indicated mRNAs. Right panels show higher magnification of embryos from adjacent left panels. Overexpression of plrg1 rescues the developmental abnormalities in the plrg1(hi3174aTg) mutants. (TIF) [file pgen.1002922.s008.tif]
