# Supplementary material for: Ccdc94 Protects Cells from Ionizing Radiation by Inhibiting the Expression of p53
Source: PLoS Genet. 2012 Aug 30;8(8):e1002922. doi: 10.1371/journal.pgen.1002922 (PMC3431329; doi:10.1371/journal.pgen.1002922)
Supplement: Text S1 — Supplemental Experimental Procedures. (A) Primers used for subcloning are listed and include the name of the target gene as well as restriction sites used for directional cloning into the pCS2+ expression plasmid. (B) The sequences of morpholinos used in this study are listed. The plrg1 morpholino was previously described [38]. (C) Primers used to examine specificity of the prp19 e3i3 morpholino are listed. See also Figure S7. (D) Primers used to sub-clone zp53 into pGEM-T-Easy for use in p53 in situ hybridization experiments are shown. See also Figure 3B. (E) Primer/probe sets used for qPCR analysis are grouped by presence or absence of shading. (PDF) [file pgen.1002922.s009.pdf]

# Supplemental Experimental Procedures:

## (A) mRNA expression constructs: Primers used for sub-cloning into pCS2+

| | |
|---|---|
| zccdc94-EcoRI-fwd: | CATGGAATTCATGTCGGAAAGAAAAGTTTTAAAT |
| zccdc94-XhoI-rev: | CATGCTCGAGCTAGTCTGAGCTGCTGTCG |
| hCCDC94-EcoRI-fwd: | CATGGAATTCATGTCGGAGCGAAAAGTATTAA |
| hCCDC94-XhoI-rev: | CATGCTCGAGTCAGTTGCTGCCGTTGCTG |
| zbcl-2-EcoRI-fwd: | CATGGAATTCATGGCTAACGAAATTAGCTATG |
| zbcl-2-XhoI-rev: | CATGCTCGAGTCACTTCTGAGCAAAAAAGGC |
| zbclxL-EcoRI-fwd: | CATGGAATTCCATGTCTTACTATAACCGAGAAC |
| zbclxL-XhoI-rev: | CATGCTCGAGTCACAGGCGTTTCTGTGC |
| egfp-ClaI-fwd: | CATGATCGATATGGTGAGCAAGGGCGAG |
| egfp-EcoRI-rev: | CATGGAATTCGACTTGTACAGCTCGTCCATG |
| zplrg1-R1-For | CATGGAATTCATGACCGAGGACGTGCAG |
| zplrg1-Xho-Rev | CATGCTCGAGCTAAAATCGCTTTCTTTTCAGAATC |
| zplrg1-mut-For4rescue | CATGGGATCCATGACAGAAGATGTCCAAAAACATTCAGTCC |

## (B) Morpholinos:

| | |
|---|---|
| translation-blocking "*ccdc94 atg*": | AACTTTTCTTTCCGACATGGTTCTC |
| splice-blocking *prp19* e3i3: | AGTCAGGTTAAAGGACTCACCCACT |
| translation-blocking *plrg1*: | AGTGCTTCTGCACGTCCTCGGTCAT (Kleinridders et al, 2009) |
| control morpholino: | ATTTGTCCCATCATTCAACGTGTTG |

## (C) Validation of prp19 morpholino specificity: Primers used to amplify prp19 cDNA

| | |
|---|---|
| prp19exon1for (F1) | ATAGCGGAGAATGGAGCTGA |
| prp19intron2rev (R2) | CCCCCATGATCAGACTGTTT |

## (D) Whole-mount in situ hybridization: Primers used for sub-cloning into pGEM-T-Easy vector

| | |
|---|---|
| zp53-R1-For | CATGGAATTCATGGCGCAAAACGACAGCC |
| zp53-Xho-Rev | CATGCTCGAGATCAGAGTCGCTTCTTCCTTC |

## (E) qPCR primers and probes (Roche Universal Probe Library)

| | |
|---|---|
| z28S probe: | Universal Probe #103 |
| z28S-for: | AAACCAACCCGGAGAAGC |
| z28S-rev: | CGCGAGATTTACACCCTCTCT |
| zapaf1 probe: | Universal Probe #123 |
| zapaf1L2 | CCTGCGCTTCCTAATGCT |
| zapaf1R2 | CTGCATTGGATATCAAAAGACCT |
| zcasp7 probe: | Universal Probe #117 |
| zcasp7L1 | AAGGAGACGTGTGCAAGAGC |

| | |
|---|---|
| zcasp7R1 | GACAGGCCTGGATGAAAAAG |
| zgapdh probe: | Universal Probe #147 |
| zgapdh-for: | CAGGCATAATGGTTAAAGTTGGTA |
| zgapdh-rev: | CATGTAATCAAGGTCAATGAATGG |
| zp53 pre-mRNA (intron9) probe: | Universal Probe #10 |
| zp53-intron9-for: | GCACAATACACCAGTGGTAAGG |
| zp53-intron9-rev: | GCAAAAACAATCAGATGCACTT |
| zp53 mRNA (prom1) probe: | Universal Probe #7 |
| zp53prom1F: | GAGGTCGGCAAAATCAATTC |
| zp53prom1R: | CACCTGGGGGCTGAATAAT |
| zp73 probe: | Universal Probe #83 |
| zp73L1 | CTTTCCAGCAGTCCAGCAC |
| zp73R1 | TTCTTGAGCAATGGAGAGTACG |
| zplrg1 probe: | Universal Probe #121 |
| zplrg1F | AAACCTATAGCGCAGGACGA |
| zplrg1R | GTATTCGGCCCGCATCTT |
| zpuma probe: | Universal Probe #18 |
| zpuma-for: | GCCTTCAGCTTGGACAGC |
| zpuma-rev: | GGACACTTCCTGTTCTGTTCCT |
